# Zoonotic and Environmental Sources of Infant Enteric Pathogen Infections Identified with Longitudinal Sampling

**DOI:** 10.1021/acs.est.5c02027

**Published:** 2025-06-23

**Authors:** Abigail P. Paulos, John Mboya, Jeremy Lowe, Daehyun Daniel Kim, Hannah C. Wharton, Faith Thuita, Valerie L. Flax, Sammy M. Njenga, Angela R. Harris, Amy J. Pickering

**Affiliations:** † Department of Civil and Environmental Engineering, 1438 University of California, Berkeley, California 94720, United States; ‡ Innovations for Poverty Action, P.O. Box 72427-00200, Nairobi, Kenya; § Department of Civil, Construction, and Environmental Engineering, 6798 North Carolina State University, Raleigh, North Carolina 27695, United States; ∥ Department of Public & Global Health, 107854 University of Nairobi, P.O. Box 30197-00100, Nairobi Kenya; ⊥ 6856 RTI International, Durham, North Carolina 27709, United States; # 118982 Kenya Medical Research Institute, P.O. Box 54840-00200, Nairobi, Kenya; ¶ Chan Zuckerberg Biohub, San Francisco, California 94158, United States; ∇ Blum Center for Developing Economies, University of California, Berkeley, Berkeley, California 94720, United States

**Keywords:** environmental microbiology, zoonotic pathogen transmission, exposure assessment

## Abstract

Many enteric pathogens that infect young children can be zoonotic, yet the exposure risk of domestic animals living in close proximity to young children is poorly understood. Here, we longitudinally measured 33 enteric pathogens in child stool, animal feces, and the household environment (*n* = 28,743 pathogen-sample observations) to investigate pathogen transmission between animals and children under two in pastoralist communities in rural Northern Kenya. Children were typically infected with 1 enteric pathogen by 3 months of age, and pathogen burden increased with age; 85% of enteric pathogens detected in child stool were also detected in animal feces. New infections in children were associated with preceding household detection of the same pathogen in soil (Odds ratio: 8.8, 95% confidence interval: 3.3–23) and on child hands (odds ratio: 5.0, 95% confidence interval 1.1–17). Regression modeling revealed transmission of pathogens from poultry, dog, and ruminant feces to household soil, and between child hands and child stool. Our results provide new evidence that domestic animals in the household environment contribute to early life enteric pathogen exposure, and that child hand hygiene could substantially prevent animal-child transmission.

## Graphical abstract

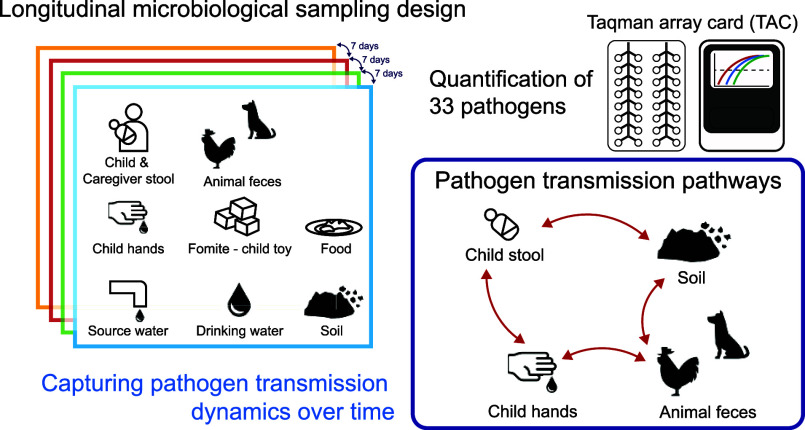

## Introduction

The annual burden of diarrhea in Africa is estimated at 1 billion cases and >500,000 deaths, with the majority of deaths occurring in children under 2 years old.^1^ The etiology of child diarrhea in Africa is diverse, including viruses (rotavirus, norovirus, adenovirus), bacteria ( pathotypes, spp., ), and protozoa (,^1,2^ Diarrheal and other enteric pathogens (e.g., helminths) also contribute to malnutrition, increasing susceptibility to future enteric infections and related morbidity and mortality.^3–5^ Fecal-oral transmission routes for enteric pathogens in settings with limited sanitation and hygiene infrastructure are complex; previous studies have detected fecal contamination in drinking water, food, soil, on hands, flies, and surfaces.^6–8^ While increased fecal contamination in drinking water and on child hands have been shown to be associated with child diarrhea, there have been limited studies that have measured contamination in other environmental reservoirs.^8^

Previous studies investigating environmental exposure pathways have mostly measured fecal indicator bacteria concentrations instead of actual pathogens. These studies often use a quantitative microbial risk assessment framework to estimate probability of illness, which requires strong assumptions about how fecal indicator bacteria relate to illness risk.^6,9–16^ Fecal bacteria levels in environmental samples are not good predictors of the presence of specific pathogens or markers of fecal contamination,^17,18^ and pathogen transmission pathways may substantially differ from fecal indicator bacteria due to differences in the fecal source and environmental fate and transport. A lack of direct measurements of viral, bacterial, protozoan, and helminth pathogens in the household environment limits our ability to elucidate key transmission pathways to young children.^19^

Exposure to animal feces is hypothesized to play a role in household enteric pathogen transmission. Prior research has found that living in close proximity to animals is associated with increased exposure to fecal contamination and increased child diarrhea.^17,20–23^ Host-specific fecal markers from animals (dogs, birds, and ruminants) were detected in multiple household reservoirs (soil, on hands, and in stored drinking water) in rural and urban Bangladesh.^17,24^ In Ecuador, identical sequence types of and atypical enteropathogenic were found in both child stool and household animals.^25^ However, measurement of pathogens in animal feces has been limited to cross-sectional studies sampling a small number of animals at the community level (e.g., not paired with human or environmental samples from the same household).^19,26^ Sampling child stool and domesticated animal feces from the same households over time is needed to understand the role of animals in early child enteric infections.

Here, we use a longitudinal prospective cohort study design to conduct extensive enteric pathogen testing of child stool, caregiver stool, animal feces, child hands, soil, food, and water from the household environment. Our goal was to identify sources of new infections in children under 1 year of age and possible environmental transmission pathways.

## Materials and Methods

### Study Site

This study was conducted between January and May 2022 in pastoralist and agro-pastoral communities in the Turkana South and Samburu North sub counties of the arid and semiarid lands regions of Kenya. The project was conducted in collaboration with the USAID Nawiri project in their program area, a USAID Bureau of Humanitarian Assistance (BHA) funded Development Food Security Activity designed to reduce persistent acute malnutrition in four counties of northern Kenya, including Turkana, Samburu, Marsabit and Isiolo. The primary occupation in the study areas is pastoralism and agro-pastoralism. Water insecurity in the area is exacerbated by droughts and flooding.^27^ For a description of access to water, sanitation, and hygiene (WASH) in the study setting, see the Supporting Information. Prior county-wide surveys in Turkana and Samburu have found low access to improved WASH, high poverty rates, high rates of animal ownership, and elevated levels of child diarrhea and acute malnutrition.^28,29^

### Enrollment and Data Collection

We enrolled children under 2 years of age as prior work suggests that the first two years of life are vital to long-term child growth trajectories and cognitive development.^30–32^ Within each sub county, we selected 10–15 villages for study based on travel feasibility (proximity to the field lab to enable processing within 6 h) and field staff safety. In each village, we enrolled all households with a child under 2 already enrolled in a separate Nawiri longitudinal study.

We enrolled a convenience sample of households identified by community health volunteers (CHVs) to have a child under 2 years of age until we reached our target numbers in each child age group. In total, we enrolled 100 households (*n* = 50 in each sub county).

We enrolled approximately equal numbers of children across four key development age groups to characterize pathogen infection profiles in these age ranges: 0–<3, 3–<6, 6–<12, and 12–<24 months (Figure 1).^33^ Target age groups were selected based on Environmental Protection Agency (EPA) child development stages^33^ and to allow for the detection of first exposures to pathogens for young children. Approximately 25 children in each target age group (0–2 months, 3–5 months, 6–11 months, and 12–23 months) were enrolled across both sub counties. Sixty children <12 months were enrolled into a longitudinal cohort and visited up to four times approximately 7 days apart.

**Figure 1:**
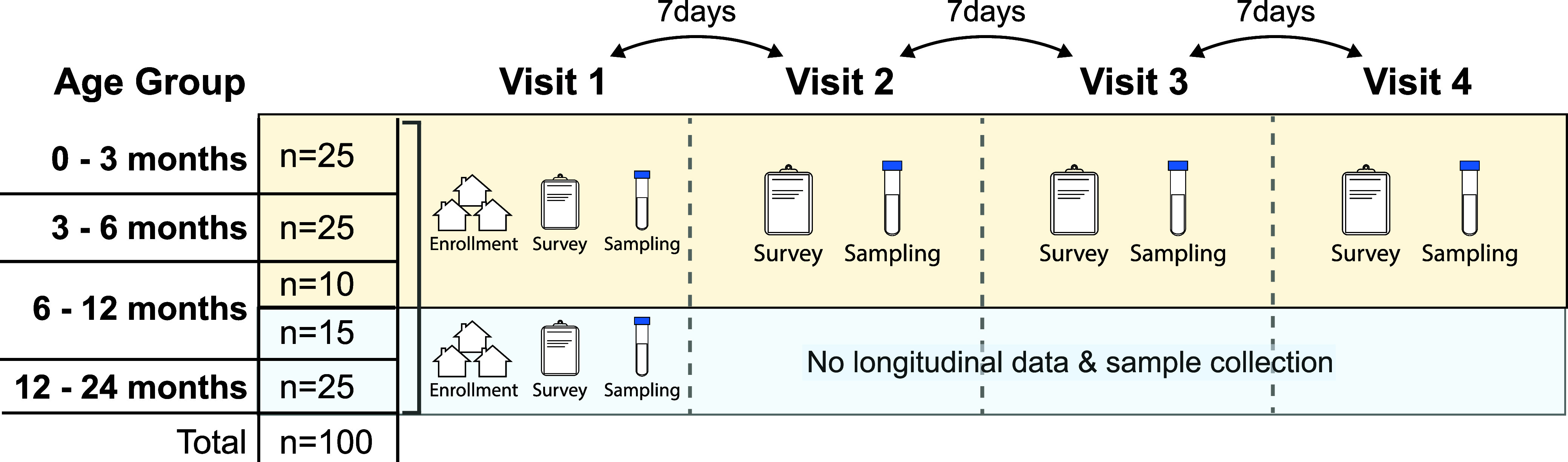
Conceptual diagram of study design. Twenty-five children were enrolled within each target age group (0 to <3, 3 to <6, 6 to <12, and 12 to <24 months). Children <6 months of age and a subset (*n* = 10) of children between 6 and 12 months of age were sampled longitudinally across four total visits with 7 days between each visit; all other children >6 months were sampled once. The sampling symbol represents sample collection of soil, drinking water, child hand rinse, fomite rinse, food, animal feces, and child and caregiver stool.

In each household, we collected the following samples: child and caregiver stool, fresh animal feces, soil, stored food, stored drinking water, and child hand rinses. Samples were analyzed using a custom TaqMan Array Card (TAC) designed to detect 33 pathogens known to cause diarrhea or other child morbidity in Kenya or other LMICs (Table S1).

IRB approval for this study was obtained from North Carolina State University (24097) and Amref Health Africa in Kenya (P1055-2021), and an in-country research permit was obtained from NACOSTI.

### Sample Collection

Stool samples were collected from the target child under the age of two years and each mother/caregiver in eligible households. Mothers were given two stool collection kits (for herself and her child) and instructed on how to fill the containers. Field staff revisited the household the following morning and up to three times in total to collect the stool samples.

We collected fresh animal feces from one of each of the following types of animals when they were available in the household of the target child: chickens, cattle, goats, dogs, donkeys, sheep, and camels. The field staff observed animals defecating and collected feces as soon as possible after defecation; otherwise, our team asked respondents to point out where the freshest animal feces was located and the sample was collected from the ground. Veterinary technicians on the field team performed rectal palpation on goats, sheep, and cattle to obtain freshly defecated animal feces when possible, approximately 75% of animal fecal collection.

The soil surrounding the entrance to the household (<2 m away from the entrance) was sampled as this location has been found to frequently contain high levels of fecal bacteria, as well as soil transmitted helminth eggs in Kenya and Tanzania during previous studies conducted by our team.^6,24,34^ Field staff identified stored weaning food to be served to children <2 years in the household and asked the respondent to provide a small amount of food in the same manner they feed their children, including serving it on a plate/bowl using any common utensils. Food types included porridge, chapati, potatoes, cassava, beans, rice, and others.

For drinking water sample collection, field staff asked the respondent to provide a glass of water as if giving it to their <2 year-old child. Water was poured directly from the glass/cup into a sterile Whirlpak bag. Field staff collected >250 mL of drinking water at each household. For hand rinse samples, field staff asked the respondent to place the target child’s left-hand into a sterile Whirlpak bag prefilled with 250 mL of distilled water and collected the sample as previously described.^35^

### Sample Processing and Analysis

All samples were preserved on ice in cooler boxes and transported to the field lab to be processed within 6 h of sample collection using the IDEXX most probable number (MPN) method with Colilert media and QuantiTray 2000s to detect and total coliform. A 100 mL aliquot of each drinking water and child hand rinse sample was processed by membrane filtration using a 0.45 μm HA filter pretreated with 2 mL of 1.25 M of magnesium chloride. Filters were preserved by submerging in 0.5 mL of Zymo DNA/RNA Shield. Aliquots of the fecal, food, and soil samples were prepared and consisted of 0.3 g of sample in 750 μL of Zymo DNA/RNA Shield. Aliquots of all samples were shipped frozen to our laboratory in the United States on dry ice.

The ZymoBIOMICS DNA/RNA Miniprep Kit (Zymo Research) was used to extract nucleic acids from all animal feces, human stool, and food samples; for these sample types, the entire aliquot of 0.3 g in 750 μl of Zymo DNA/RNA shield was processed. Lysis buffer volume was increased by 2.5x to 1200 μL to ensure complete lysis in all samples. For soil samples, we used the RNeasy PowerSoil Total RNA Kit with the DNA Elution Kit add-on (Qiagen) to coextract RNA and DNA; the input mass was 2 g. For both kits, we eluted in 100 μL and stored extracts at −80 °C until TAC analysis.

We designed a custom TaqMan Array Card (TAC; ThermoFisher) containing enteric pathogen targets (Table S1). The TAC is a platform for quantitative real-time PCR that enables simultaneous detection of up to 48 targets per sample in one run. We selected enteric pathogen targets associated with diarrhea or other diseases of child health significance, previously detected in Kenya or similar settings. In brief, we used 2-step reverse transcription followed by TAC to detect and quantify pathogens in nucleic acid extracts. Because of low nucleic acid content, food, child hand rinse, and drinking water samples were subjected to preamplification using the same set of primers on the custom TAC card between RT and TAC analysis (see Supporting Information for details). Primers and probes for assays included on the custom TAC are listed in Table S1, standard curve efficiencies in Table S2, sample limits of detection in Table S3, and gBlock sequences used for the standard curve in Table S4.

### Statistical Analyses

New infections were estimated for children enrolled in the longitudinal cohort and were defined as (1) no carriage of the pathogen in the prior round and (2) carriage in the current round. Pathogen presence in environmental samples were lagged by one visit and we calculated risk ratios for new infection in children given pathogen presence 7 days prior in (1) animal feces, (2) mother stool, (3) soil, (4) drinking water, and (5) any environmental sample (soil, child hands, food, or water). Odds ratios were estimated using Fisher’s exact method with confidence intervals. Odds of pathogen codetection were calculated between all pairs of sample types using Fisher’s exact method for confidence interval estimation.

To explore overlap between environmental pathways, we also calculated odds ratios for (1) leading pathogen contamination, defined as the same pathogen present in another sample type at the prior visit, and (2) pathogen codetection, defined as the same pathogen present in multiple sample types on the same visit. Using these odds ratios, we created an aggregated pathogen transmission visualization which displays the number of connections across the 20 pathogens with significant leading pathogen odds ratios (Figure 5). We also created a graph for each pathogen representing pathogen overlap across sample types, where each circle is a sample type and each arrow indicates an odds ratio with *p* < 0.05 (Figure S3). The width of each arrow represents the magnitude of the odds ratio with the width increasing with odds ratio. Nodes which had no connections with other nodes were excluded. Three categories of animal feces were considered: ruminants (cows, sheep, and goats), chickens, and dogs. The open source tool graphviz was used to generate graphs of pathogen overlap.^36^

Analyses were conducted in R version 4.2.3.

## Results

### Pathogen Detection and Quantities

A total of 100 households were enrolled into the study, including 60 into the longitudinal cohort; detailed participant enrollment and sample collection numbers are listed in Tables S5 and S6. We generated 28,743 observations (33 pathogens measured in 871 samples). Most (80%) samples contained at least one pathogen. The most frequently detected pathogens across all sample types were (35%), enteroaggregative (EAEC: 30%), enteropathogenic (EPEC: 29%), enterotoxigenic (ETEC-LT: 22%), and Shiga toxin-like (STEC: 19%) (Figure 2, Table S7). In child stool, the most common pathogens were EAEC (56%), enterovirus (47%), EPEC (36%), (20%), adenovirus 40/41 (15%), ETEC-LT (15%), and norovirus GII (12%). Overall, 87% of child stool samples contained at least one pathogen. In the longitudinal cohort, all 56 children were infected with one or more pathogens in at least one visit. Children overall were infected with a median of 4 pathogens, and pathogen carriage increased with child age (*t* tests; see Figure 2): a median of 1 pathogen for 0–2 months, 2 pathogens for 3–5 months, 3 pathogens for 6–11 months, and 5 pathogens for 12–23 months. Of the 20 different pathogens detected in child stool, 17 of these were also detected in animal feces, 17 in soil, 10 in drinking water, 5 in food, 17 on child hands, and 16 in caregiver stool.

**Figure 2:**
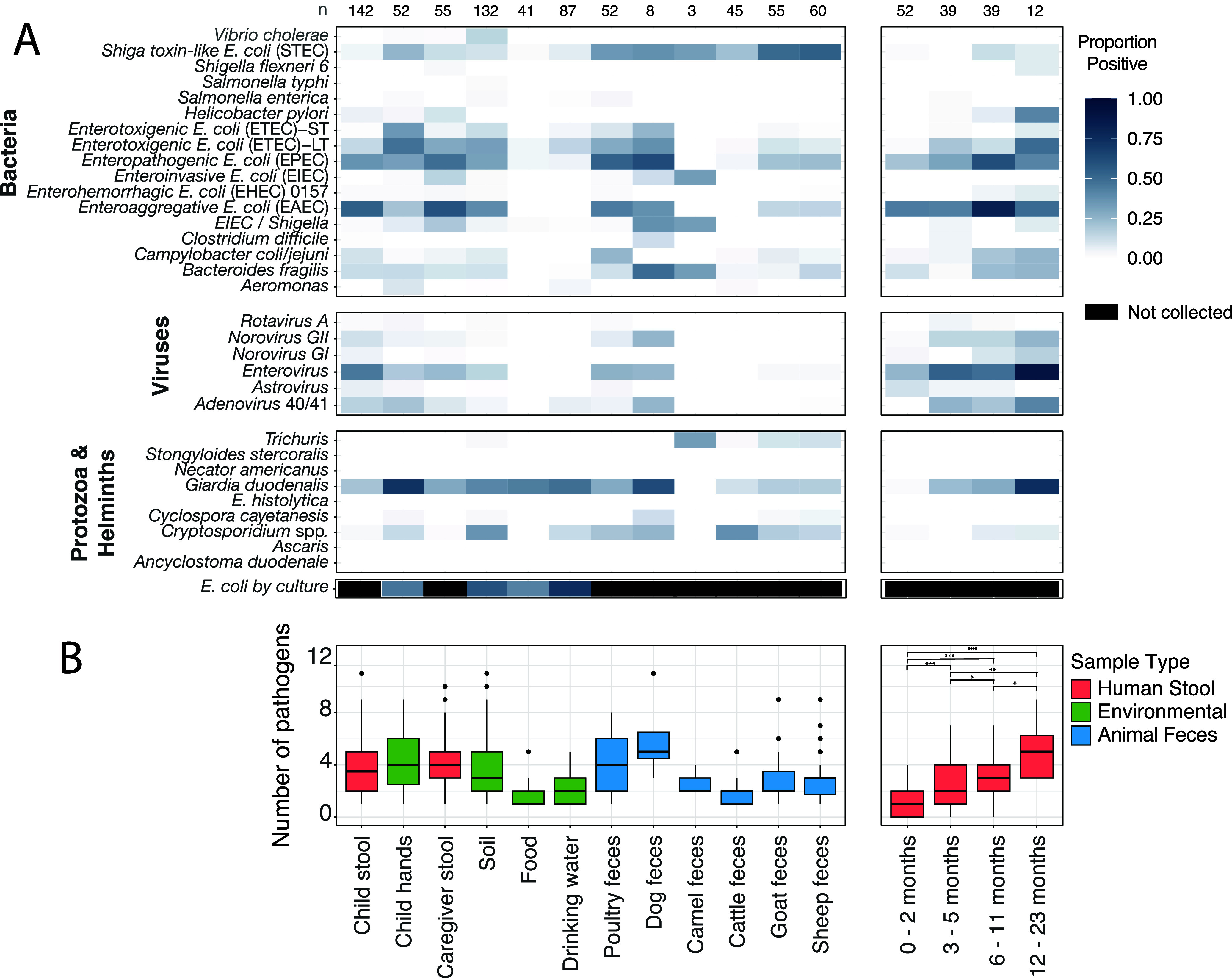
Enteric pathogen profiles across sample types: (A) proportion of samples positive for each pathogen across sample types (left) and in child stool by age (right). Sample sizes for each group are indicated at the top of the figure. (B) Number of pathogens (out of a maximum of 33 pathogens) present by sample type and child age. * *p* < 0.05 ** *p* < 0.01 *** *p* < 0.001

Although not a main objective of this study because of the limited number of children enrolled with diarrhea, we find that rotavirus was significantly and positively associated with caregiver-reported child diarrhea at the time of sample collection (Table S8); for diarrheal prevalences in the study, see Table S9.

Animal feces carried a range of human enteric pathogens. To assess the diversity of pathogens in each sample type, we summed the number of different pathogens present in each sample out of a maximum of 33 pathogens (Figure 2B). Dog feces carried the greatest number of unique pathogens (median = 5); poultry feces had a median of 4 pathogens. Ruminant feces carried the smallest number of unique pathogens among all animal types (cattle: 2, goats: 2, and sheep: 3). Poultry and dogs were frequently infected with pathogenic (poultry: 76%, dogs: 92%; Figure S1), and infections of (27%, 69%), enterovirus (27%, 23%), and nonparvum and nonhominis spp. (24%, 38%) were also common. Cattle also frequently carried STEC (22%), (11%) and spp. (38%). Goats and sheep were most commonly infected with STEC (goats: 51%, sheep: 53%), EPEC (23%, 23%), spp. (18%, 15%), and (19%, 18%).

Food contained a median of 1 enteric pathogen while drinking water contained a median of 2 pathogens; for both sample types, the pathogen most commonly detected was (food: 45%, water: 46%). Child hands carried a median of 3 pathogens per child, primarily (74%) and forms of pathogenic (EAEC: 14%, EPEC: 24%; ETEC-LT: 46%; ETEC-ST: 27%); 77 of 92 child hand samples contained at least one pathogen. Soil contained a median of 3 pathogens, primarily (42%), spp. (35%), and forms of pathogenic (EAEC: 42%, EPEC: 37%, ETEC-LT: 36%, ETEC-ST: 15%). For pathogen quantities by sample type, see Figure 3.

Approximately 75% of stored drinking water samples contained culturable , and over a third of all other sample types tested positive for by culture: 44% of food, 48% of child hands, 45% of fomites, 62% of soil. Pathogen concentrations in all samples were calculated with TAC results using a standard curve (Figure 3; Table S2). Across food, soil, drinking water, and child hands, we found few associations between pathogen and culture presence or concentrations using Spearman’s correlation tests: concentrations in culture were associated with EIEC on hands (*r* = 0.47, *p* = 0.05), ETEC-LT in drinking water (*r* = 0.56, *p* < 0.01), and norovirus GII in soil (*r* = 0.42, *p* = 0.01) and on hands (*r* = 0.47, *p* = 0.05; Table S10). concentrations measured by TAC (*uidA* gene) were associated with on hands (*r* = 0.68, *p* < 0.01) and in drinking water (*r* = 1.00, *p* < 0.01), astrovirus in chicken feces (*r* = 0.78, *p* < 0.01), and EPEC in goat feces (*r* = 0.71, *p* < 0.01; Table S10). For sample numbers processed for culture, see Table S6.

**Figure 3:**
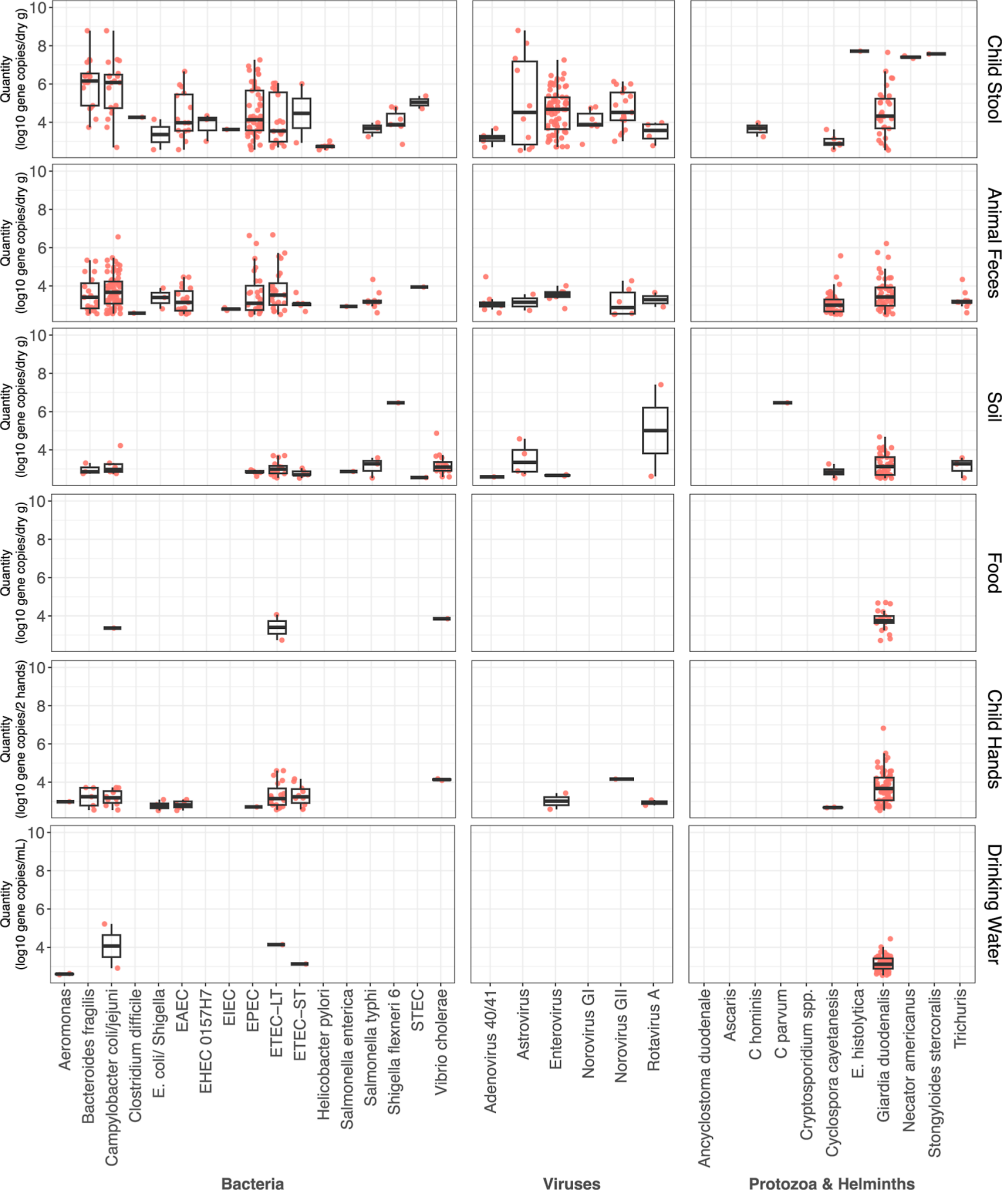
Distribution of quantities among positive samples measured by TAC by sample type (*n* = 732 samples).

### Longitudinal Analysis of Environmental Pathways for New Infections

In total, we detected 33 new infections across 10 of the 33 measured pathogens, with new infections detected in 20 of the 56 children (36%) sampled longitudinally (4 children were unavailable for follow up visits). Children with new infections had a median of 1 new infection; 7 of 20 children were newly infected with more than one pathogen in the same visit and 3 of 20 children were newly infected across multiple sampling rounds. The number of new infections decreased with child age; we observed 15 in children 0–2 months (73% of children in this age group), 12 in 3–5 months (60%), and 6 in 6–11 months (50%). Children were most often newly infected with EPEC (*n* = 8), EAEC (8), enterovirus (5), norovirus GII (4), (2), and adenovirus (2). Pathogens responsible for 1 new infection included STEC, ETEC-LT, astrovirus, and / Overall, across the 33 new infections, we identified the same pathogen in any type of pathway (environmental samples, animal feces, or mother stool) on the prior visit in 16 (48%) cases.

Pathogen detection in soil in the prior sampling round was associated with new infections (odds ratio [OR]: 8.8, 95% CI: 3.3–23, *p* < 0.01; Figure 4), as was pathogen detection on child hands (OR: 5.0, 95% CI: 1.1–17, *p* = 0.02). When aggregated across all environmental pathways (hands, soil, water, and food, when available), we find that pathogen detection in environmental pathways was associated with new infections (OR: 6.1, 95% CI: 2.7–14, *p* < 0.01). We aggregated results of all animal feces types (due to limited power to investigate by specific animal host), yet we found no statistically significant association between new infections and pathogen presence in animal feces on the previous visit (OR: 2.2 95% CI: 0.6–6.3, *p* = 0.15). We found no associations between new infections in child feces and food, water samples, or mother stool as there was limited overlap in the specific pathogens found in child stool and these sample types; the same pathogen which newly infected a child was detected in 0 food samples, 2 water samples, and 2 mother stool samples 7 days prior. We also assessed the odds of child new infection given pathogen detection in each sample type either on the prior visit or the same visit; we find that pathogen detection in animal feces and mother stool are associated with new infections (Figure S2).

**Figure 4:**
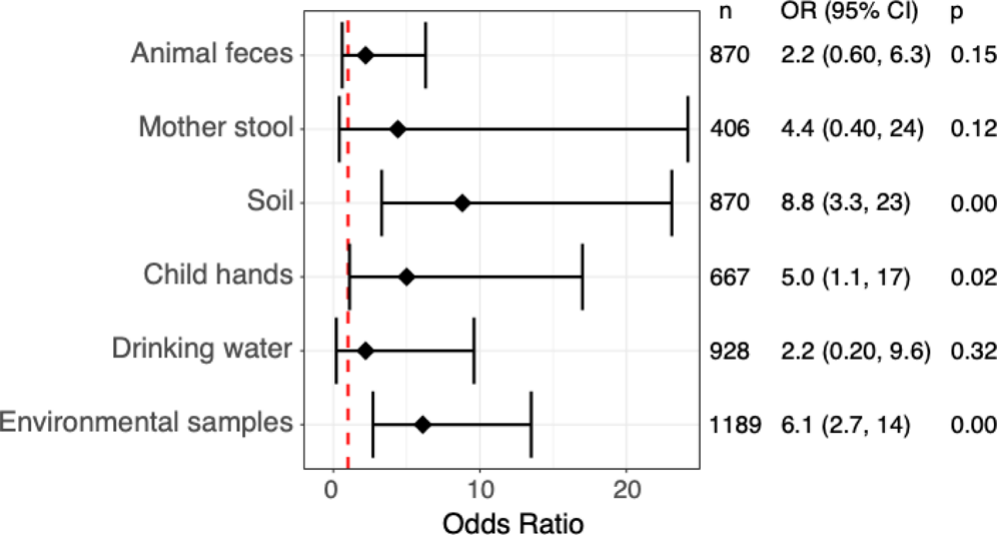
Odds ratios of pathogen detection in animal feces or environmental samples and subsequent new infection with the same pathogen in children. Bars represent 95% confidence intervals. The dotted red line indicates an odds ratio of 1. The environmental samples category includes hands, soil, water, and food. Food alone is not displayed because of large uncertainty around the point estimate.

Food sample collection in the household was limited as few households had food available at the time of collection, or caregivers solely breastfed their child; only *n* = 40 total samples were collected, compared to n = 116 for drinking water and *n* = 163 for soil. However, we estimated the odds of pathogen detection in food given detection in soil or animal feces, and we find that the odds of pathogen detection in food was 13 times higher when that pathogen was present in either soil or animal feces on the same visit (OR: 13, 95% CI: 7.5, 23).

### Pathogen Transmission Graphs

Across 13 of 20 pathogen graphs, there was a connection between child hands and child stool, highlighting the role that hands play in transmission of these pathogens (Figures 5 and S3). Of the pathogen graphs with an exposure associated with subsequent child stool infection, hands were the most common (6 of 10), followed by soil (4 of 10), chicken feces (4 of 10), caregiver stool (1 of 10), dog feces (1 of 10), and food (1 of 10). Animal feces had a connection to soil in 9 of 20 pathogen transmission graphs, and a connection to any other environmental pathway in 16 of 20 graphs. Detection in ruminant and chicken feces preceded caregiver infection for , *EIEC*/*Shigella*, *EPEC*, and *STEC*. Overall, pathogens were shared across 5 or more sample types for 16 of 20 pathogens. For 13 pathogens, no significant relationships were found between any sample types due to rare or no detections.

**Figure 5:**
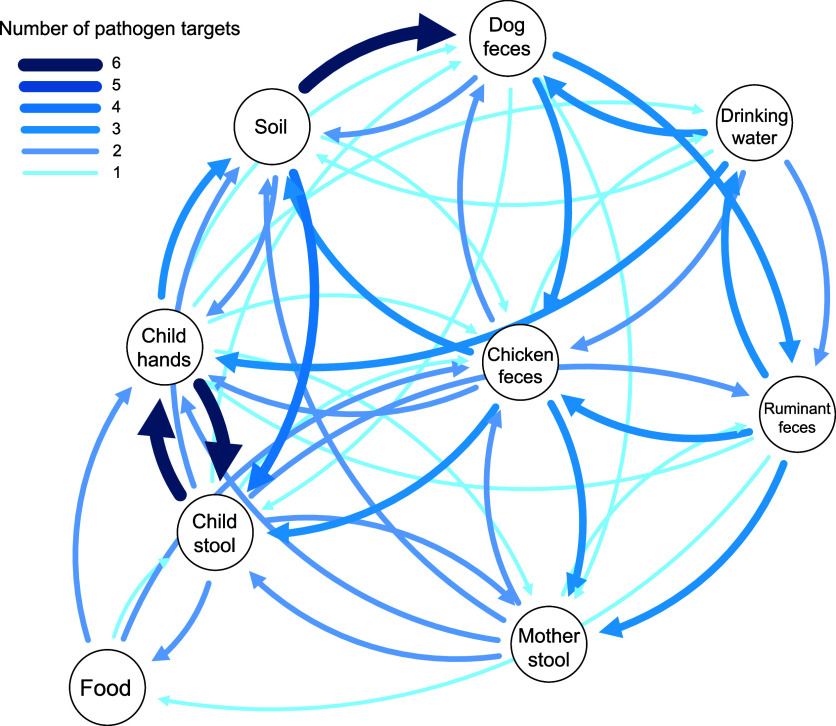
Visualization of temporal associations of the same pathogen present in hosts and environmental samples. Circles represent the sample type. Arrow widths represent the number of pathogens for which detection in the sample type was associated with detection in the receiving sample type on the next visit within the same household (logistic regression models, association is included if odds ratio is significant *p* < 0.05; *p*-values not adjusted for multiple comparisons).

## Discussion

Our results provide the first longitudinal evidence that animal feces are an important source of initial and early life (<12 months) child enteric infections. We found substantial overlap of pathogens in child stool and animal feces; 85% of pathogens detected in child stool were also detected in animal feces. Detection of a pathogen in animal feces within a household was significantly associated with child infection with the same pathogen (Figure S2). Child hands and soil were important environmental transmission routes for infant infections, particularly for pathogens originating in chicken feces (Figure 5). Detection of adenovirus, astrovirus, , , and several pathotypes on a child’s hands frequently preceded child infection with these pathogens. Soil contamination with astrovirus, norovirus, and pathotypes often preceded child infection. These findings highlight that hands and soil are transmission pathways for child-animal pathogen exchange within the domestic environment. As livestock are a critical source of household income and nutrition for young children,^37^ our results indicate a need for improved community and household infrastructure and practices to separate children from animal feces.

This study generated new evidence of animal fecal shedding of a wide range of human enteric pathogens. Some of these pathogens, such as pathogenic , spp., and , are known or suspected to be zoonotic, while others such as enterovirus, adenovirus, and norovirus are not typically considered zoonotic.^21^ Animal feces were widespread in the household environment, even in households that did not report owning animals (see results on household characteristics the Supporting Information). For both pathogens typically considered zoonotic and those that are not, human feces are one potential initial source of these pathogens in animals. Poor human waste management in the study area may enable animal consumption of human feces and result in reverse zoonotic transmission (transmission from humans to animals).^38^ Notably, we find that detection of pathogens in child stool is associated with subsequent contamination of soil with the same pathogen, suggesting that poor management of child waste could also contribute to pathogen proliferation in the environment and, potentially, animal infections. Provision of safely managed sanitation could prevent reverse zoonotic transmission, yet prior work has found that provision of basic sanitation alone is not adequate to reduce enteric pathogen infection prevalence in children under 5 years.^39^ Further, many pathogens frequently infecting children in these settings are known to be zoonotic, and transmission within animal species can be sustained even without exposure to human feces.^40–42^ Safely managed sanitation infrastructure that integrates both animal feces and child feces management may be necessary to prevent both zoonotic and reverse zoonotic transmission.

The household environment in our study site was widely contaminated with across all sample types. is a leading cause of diarrhea in Africa, with an estimated 36,116 annual cases per 100,000 people.^1^ The prevalence of infections in children in our study (20%) exceeded that of Kenya overall (9%),^43^ but was lower than the prevalence found in healthy children in other settings.^44^ Prior to this study, contaminated food and drinking water were well-known transmission pathways for .^45^ Here, we observed animal feces contaminating household soil and child hands with . Nearly half of all soil samples contained , compared to 18% of samples in an urban community in Kisumu, Kenya.^46^ Contamination with on child hands (74%) was much higher than has previously been reported; a meta-analysis found that less than 2% of mother hands carried .^17,47^ has been previously detected in the feces of both livestock and household pets, which aligns with our observation of in >50% of dog and >25% poultry feces.^21^ Notably, the prevalence of in dog feces in our rural study site was approximately 3 times higher compared to dogs in an urban community in Kenya.^48^ Detection of on child hands and in dog feces temporally preceded child infection. Having a dirt floor and chicken ownership were both risk factors for infections in children under 2 in previous studies.^49,50^ Our findings indicate animal feces as an important source of child infections through child hand contact with soil in rural, low-income settings.

Many prior studies have used as an indicator of fecal contamination in environmental reservoirs, and studies measuring bacterial, viral, protozoan, and helminth enteric pathogens have been limited.^19,51^ However, we found that is generally not associated with enteric pathogen detection in soil, food, drinking water, or on child hands. Of the most commonly detected pathogens in this study (EPEC, EAEC, ETEC, STEC, enterovirus, , adenovirus 40/41, and norovirus GII), detection in an environmental pathway was only associated with detection of ETEC-LT in drinking water and norovirus GII in soil and hands. Accordingly, detection in these environmental reservoirs does not imply the presence of any specific enteric pathogens. Published studies conducting quantitative microbial risk assessments (QMRAs) often infer specific pathogen quantities in environmental reservoirs by assuming a ratio between measured and other pathogens.^52^ Our results suggest that this approach will not provide valid estimates of enteric pathogen levels, as there was no association between levels and most pathogens across multiple environmental media. Other studies using TAC have also found variable pathogen detection despite presence, as in Capone et al.^51^

Reported diarrheal rates in our study were high, and the pathogens we detected among children suggest different diarrhea etiologies in this setting than reported in prior studies. In the Global Enteric Multicenter Study (GEMS), the pathogens associated with diarrhea in children <12 months in Nyanza Province, Kenya were found to be rotavirus (22% of cases), spp. (9.9%), ETEC (8.8%), and adenovirus 40/41 (8.5%). For children between 12 and 24 months of age, the most important pathogens were EIEC/ (15.4%), rotavirus (14.5%), ETEC (9.5%), and spp. (6.3%).^2^ A recent meta-analysis found that in East Africa, the pathogens responsible for the greatest diarrheal cases in children under 5 were EAEC, , ETEC, and norovirus, while those responsible for the greatest number of deaths were EIEC, rotavirus, and spp.^1^ In this study, the prevalence of these pathogens in child stool was low, with the exception of EAEC, , and adenovirus 40/41; however, diarrheal prevalence was high, and we found that child diarrhea was associated with rotavirus. Notably, GEMS was located in a region with different climate, culture, and animal husbandry practices than our study site, all of which may impact prevailing pathogen profiles. Prior research has found that practices of nomadic and pastoral peoples can increase the risk of zoonotic enteric parasite transmission;^53^ we hypothesize that these practices may also influence the types of enteric pathogens present and the key pathways for transmission.

Diarrhea has been associated with an increased risk of acute malnutrition in young children,^54–56^ as previously demonstrated in the USAID Nawiri longitudinal study in Samburu and Turkana Counties.^57,58^ The high prevalence of diarrhea in this study (>30% 7 day prevalence), and particularly the association we find between diarrhea and rotavirus, the most common cause of fatal diarrhea in children,^59^ could be contributing to the high prevalence of wasting in these two counties. Future research examining how early life enteric pathogen exposure and infection contributes to wasting would be valuable to determine the potential for interventions that prevent pathogen transmission to reduce wasting.

This study has several limitations. Sample collection occurred during the dry season, and pathogen transmission pathways could be different during the rainy season and periods of heavy precipitation. The incubation time from initial exposure to symptomatic illness and/or pathogen shedding in child feces varies by pathogen, but sample collection was standardized to occur 1–2 weeks apart to capture most incubation periods. Heterogeneity of pathogen concentrations in the environment could impact our measurements, leading to an over- or underestimate of pathogen prevalence in these sample types overall;^60^ in general, we collected the largest sample mass possible and homogenized the sample before aliquoting to reduce the effects of environmental heterogeneity. While qPCR assays have the advantage of uniquely identifying pathogens, they are unable to identify if the exact same strain is present across samples, as whole genome analysis can. A key limitation of this study is the sample size; we monitored 60 children longitudinally for approximately 4 weeks. Future studies incorporating larger sample sizes and long-term monitoring would be beneficial to further elucidate transmission pathways. Incorporating a larger sample size in a study repeatedly measuring pathogens in animal feces, the environment, and child stool would provide evidence on the specific animal species and pathogens transmitted zoonotically. Notably, our results document transmission in a specific setting; similar studies among other populations would be valuable to demonstrate transmission routes in different settings and help us identify important contextual features that influence transmission dynamics. However, this study is the first to apply longitudinal monitoring of a wide range of pathogens by qPCR across child and caretaker stool, animal feces, and investigate a comprehensive set of environmental transmission pathways.

Here, we demonstrated that children become infected with pathogens shortly after birth despite reported exclusive breastfeeding. In this setting, the youngest children (<6 months of age) quickly became infected with pathotypes, enterovirus, and . Children under 6 months of age, and especially children under 3 months, are often excluded from studies of pathogen infection.^12,61^ We detected significant pathogen overlap between child hands and child stool, suggesting that hand contamination often precedes child infection. Other studies have reported that hand-to-mouth contacts can be an important transmission pathway for children over 3 months of age,^9,14,18,62,63^ and we extend these findings to those under 3 months of age. Typically, community hand hygiene campaigns focus on caregiver hand hygiene while young (<2 years) child hand hygiene is often left out of behavior change messaging. Altogether with previous evidence, our findings suggest that infant hand hygiene could substantially prevent early life pathogen infections, including zoonotic pathogens.

## Supplementary Material





## Data Availability

The raw data (deidentified) and code used for analyses are available at https://github.com/abharv52/infant_enteric_pathogen_transmission.

## Notes

Additional details on methods and household characteristics (PDF)
EMMI_final_vs3 (XLSX)
