# Supplementary material for: Zoonotic and Environmental Sources of Infant Enteric Pathogen Infections Identified with Longitudinal Sampling
Source: Environ Sci Technol. 2025 Jun 23;59(26):13181–91. doi: 10.1021/acs.est.5c02027 (PMC12243083; doi:10.1021/acs.est.5c02027)
Supplement: Supplementary file 1 [file es5c02027_si_001.pdf]

Supporting Information

Zoonotic and environmental sources of infant enteric pathogen infections identified with longitudinal sampling

Abigail P. Paulos[1ϴ], John Mboya[1,2ϴ], Jeremy Lowe[1,3], Daehyun Daniel Kim[1], Hannah C. Wharton[1], Faith Thuita[4], Valerie L. Flax[5], Sammy M. Njenga[6], Angela R. Harris[3*], Amy J. Pickering[1,7,8*]
Affiliations:
[1] Department of Civil and Environmental Engineering, University of California, Berkeley, CA 94720, United States
[2] Innovations for Poverty Action, P.O. Box 72427-00200, Nairobi, Kenya
[3] Department of Civil, Construction, and Environmental Engineering, North Carolina State University, Raleigh, NC 27695, United States
[4] Department of Public & Global Health, University of Nairobi, Kenya
[5] RTI International, Durham, NC 27709, United States
[6] Kenya Medical Research Institute, P.O. Box 54840-00200, Nairobi, Kenya
[7] Chan Zuckerberg Biohub, San Francisco, CA 94158, United States
[8] Blum Center for Developing Economies, University of California, Berkeley, Berkeley, CA 94720, United States

[ϴ] AP and JM contributed equally to this paper.
* corresponding authors: pickering@berkeley.edu, aharris5@ncsu.edu

Number of Pages: 33

Text: 6

Tables: 12

Figures: 3

Methods
All samples were stored in a cooler on ice until transport to the field laboratory.

Household survey
In each household visited, we conducted a household survey with the child's caregiver over 16 years of age to collect information on child health, demographics, water, sanitation, and hygiene (WASH) status, and child behavior. The child health portion of the survey was repeated at each longitudinal visit in the longitudinal cohort.

Sample Collection
Stool sample kits consisted of a 50ml sterile container, gloves, aluminum foil, and a spatula. Animal fecal samples were collected with sterile scoops into sterile 50 ml containers.

For soil sample collection, field workers marked a 30 cm by 30 cm area using a disposable sterile stencil and scraped the top layer of soil within the stencil into a sterile Whirlpak bag using a sterile disposable plastic scoop; the sample area was scraped once vertically and once horizontally to collect approximately 50 g of soil. Field staff prioritized sampling locations that were shaded.

Food was scooped to fill as much of a 50mL sterile plastic tube as possible using a sterile spoon attached to the lid of the tube.

For hand rinses, the hand was massaged from the outside of the bag for 15 seconds, followed by 15 seconds of shaking. The same procedure was repeated with the right hand in the same bag, and the rinse water was preserved in the Whirlpak bag

Dry Weight Estimations
The dry weight and percent moisture in soil and food samples were determined by weighing out 5g of each sample and drying in a drying oven at 110°C for 24 hours. Weights of dried samples were recorded and used to estimate the percent moisture in the samples.

IDEXX E. coli culture
Culturing E. coli from environmental and fecal samples
Stored water, source water, hand rinse, and fomite rinse samples were processed by decanting 100 mL of sample directly into IDEXX trays. Food and soil samples required a homogenization step before processing with IDEXX. Four grams of each sample were placed into a 50 mL falcon tube with 40 mL distilled water, shaken by hand for 30 seconds, and vortexed for 2 minutes. One mL of this solution was mixed with 99 mL sterile water and the resulting 100 mL were processed with IDEXX. Samples were incubated at 35°C for 24 hours to quantitatively enumerate *E. coli* and total coliforms. Field and lab blanks were processed each day, and 5% of samples were processed in duplicate for quality assurance.

All samples were preserved on ice in cooler boxes and transported to the field lab to be processed within 6 hours of sample collection using the IDEXX most probable number (MPN) method with Colilert media and QuantiTray 2000s to detect *E. coli* and total coliform. The IDEXX Quanti-Tray 2000s used for *E. coli* enumeration in this study contains 49 large wells and 48 small wells (10% the volume of large wells) and can quantify in the range of 1-2,419 colonies; input is 100 mL. After incubation for 24 hours, each well individually fluoresces if *E. coli* is present. Quantities of *E. coli* are calculated using a most probable number (MPN) approach based on the number of large and small wells that fluoresce.

The range of quantification for drinking water, source water, fomite, and hand rinse samples is 1 - 2,419 colonies. For soil and food, the range of quantification is 40 - 96,760 colonies because of soil dilution.

Field and lab blanks were processed each day, and 5% of samples were processed in duplicate for quality assurance.

Field blanks consisted of 100 mL of sterile water poured into WhirlPak bags that were transported to and from the field along with other samples. Following drinking water sample collection, enumerators were instructed to open and close the field blanks in the same manner as the main samples. Any *E. coli* detected in field blanks would indicate cross-contamination during sample collection and transport and samples collected from that enumerator the same day would be discarded and recollected.

Laboratory blanks consisted of 100 mL sterile water processed alongside field samples for IDEXX. The sterile water was poured directly into the IDEXX trays after all other samples were processed. *E. coli* detection in laboratory blanks would indicate cross-contamination within the laboratory.

Aliquoting for molecular analysis

Fecal, food, and soil samples were vortexed to evenly distribute samples in the preservative. Aliquots were stored in 2mL tubes at -20°C in the local field lab before being shipped to Kenya Medical Research Institute (KEMRI) on dry ice for storage at -80°C, then shipped on dry ice to UC Berkeley for enteric pathogen molecular analysis. Water and hand rinse filters were stored in 5mL tubes with Zymo RNA/DNA shield at -20°C until transfer to KEMRI, where they were stored at -80°C.

QA/QC

Detection of total *E. coli* by the *uidA* assay on the TaqMan Array Card was used as an endogenous process control to confirm extraction success for fecal and soil samples. For food, drinking water, and hand rinse samples (which would not be expected to universally contain *E. coli)*, we spiked in 10 uL of the TaqMan Universal RNA Spike In/Reverse Transcription (Xeno) Control (ThermoFisher) during lysis and measured using the TaqMan Xeno assay on the TaqMan Array Card. An extraction was considered successful if *E. coli* (fecal and soil samples) or Xeno (food, drinking, and hand rinse samples) amplified with a Ct below 40.”

One extraction blank was included in each batch of extractions, and one no-template control (NTC) consisting of molecular grade water was included in TAC per day. Extraction and TAC blanks were considered successful when there was no amplification in any assay. If any amplification occurred in the extraction blank, samples were re-extracted; if amplification occurred in TAC, all corresponding samples were rerun. Field and laboratory blanks were tested in the field laboratory by IDEXX colilert, and only samples with negative corresponding field blanks were included in the study.

Reverse transcription and TAC
Reverse transcription was conducted using the SuperScript IV VILO kit. The reaction consisted of 4 µL of SuperScript mastermix, 7 µL of DNA/RNA free water, and 9 µL of template (sample). Reverse transcription (RT) was performed using the Invitrogen SuperScript IV VILO kit with the following cycling parameters: 25°C for 10 min, 50°C for 10 min, and 85°C for 2 min. Samples were then brought to 4°C before further processing. The RT output was diluted to a total volume of 55 µL and mixed 1:1 with 55 µL of Applied Biosystems TaqMan Fast Advanced Master Mix. 100 µL of this solution was loaded onto the TAC and ran with the following cycling parameters: 50°C for 2 min, 92°C for 10 minutes, and 50 cycles of 95°C for 1 second and 60°C for 20 seconds. A sample with a Ct value below 40 was considered positive for all samples (both with and without preamplification).

Preamplification
Food, child hand rinse, and drinking water samples were subjected to preamplification prior to analysis to increase the ability to detect nucleic acids. For preamplification, we used a custom preamplification pool using the primers in our TACs (Life Sciences, USA). Reactions consisted of 20 µL of template, 10 µL of preamp pool, and 10 µL of the TaqMan Fast Advanced mastermix. Cycling parameters were 95°C for 2 min, 14 cycles of 95°C for 15 s and 60°C for 2 min, and 99°C for 10 min. Following preamplification, 10 µL of the total reaction were used as input into the TAC reaction mixture. A Ct value below 40 was considered positive. One NTC was run through preamplification and TAC per day. Extraction blanks for food, child hand rinse, and drinking water samples were put through preamplification.

TaqMan Array Card standard curve
We used three custom gBlock DNA sequences (IDT, Iowa, USA) as template for our standard curve. Each gBlock was resuspended and all three mixed at equal concentration to create one master standard curve. We ran a 7-point standard curve on one TaqMan Array Card (TAC) with concentrations ranging from 10 gc/rxn to 10^7gc/rxn. We repeated the standard curve across a total of 6 TACs distributed throughout the sample processing period. A master standard curve was fitted to the results for each target using a linear mix//ed effects model with batch effects for card (**Table S2**). Efficiencies ranged from 96 – 118% with a median of 108%. Standard curves were used to calculate pathogen concentrations in all sample types. For food, hand rinse, and water samples, a 2^{14} reduction in estimated quantity was applied to account for the 14 cycles of

preamplification. To estimate quantities for pathogens with multiple targets, the quantity of the target with the greatest value was used.

Limit of detection calculations
The limit of detection (LOD) for each assay was defined as the lowest point on the standard curve where >50% of replicates amplified. The LOD for each sample type was calculated from each assay LOD using the dilution factor throughout the extraction, RT, and TAC process.

Inhibition Testing
Inhibition testing was conducted on soil and fecal samples, as these sample types would be the most likely to be inhibited. C_q values were compared between the raw and diluted (1:10, 1:20, and 1:50) sample. Samples were considered to be inhibited if the difference between the theoretical and actual C_q difference exceeded 1 cycle. The theoretical C_q difference for a raw sample *versus* a dilution is 3.32 cycles (1:10 dilution), 4.32 cycles (1:20 dilution), and 5.6 cycles (1:50 dilution).

Poverty line calculations
We used the Kenya Poverty Probability Index (PPI) to calculate the probability of each household falling below the poverty line and the food poverty line.[1] Questions from the PPI index were included in our household surveys, and probabilities for poverty calculated using the methods described in the PPI report.

Results

QA/QC
Samples without amplification in the *uidA E. coli* gene (human stool, animal feces, and food) or the Fisher endogenous RNA control (soil, drinking water, and hand rinses) were reprocessed and if still not positive for the above controls, excluded from the analysis. In total, 33 of 871 samples were excluded. All field, laboratory, and extraction blanks were negative for total *E. coli*; NTCs in TAC were negative for all assays. Inhibition testing found no evidence of sample inhibition so undiluted samples were processed in TAC (**Table S17**).

Household Enrollment and Characteristics
In total, we enrolled 100 households with a child under 2 years of age (**Table S13**). Approximately 25 children were enrolled in each of the age groups: 0-2 (24 households), 3-5 (22), 6-11 (30), and 12-23 months (24). We conducted structured observations in 56 households and enrolled 60 households in the longitudinal cohort. Household surveys conducted as part of this study agree with prior surveys. We find that 64% of households fall below the Kenya poverty line and 66% below the Kenya food poverty line, indicating probable food insecurity.[1] Eighty-six percent of households reported owning livestock, most commonly goats (83%), chickens (57%), dogs (49%), and cattle (42%). Animal feces were observed in every household, primarily sheep or goat (n=60 of 88 observations), poultry (33), cow or buffalo (17), and dog or cat (14). Ninety-three percent

of households lacked electricity access and 71% of child caregivers did not complete primary school.

WASH Access
Access to water, sanitation, and hygiene (WASH) was limited in the study regions. Fifty-nine percent of households lacked access to any type of latrine, and only 4% reported having a handwashing station available. Latrine types used were covered pit latrines with slab (15) and without slab (9), and uncovered pit latrines with slab (4) and without slab (10); 2 households reported using a ventilated improved pit (VIP). The primary drinking water sources were piped water (n=50) and boreholes (36), with the remaining households relying on springs (5), dug wells (5), surface water (2), and vendor water (2). Sixty-six households reported that their drinking water source was not available when needed at least once in the last two weeks; 50% of these cases were due to the source running out of water, 8% due to lack of electricity or fuel, and the remaining cases for various reasons. Reported one-way walk times to the primary water source ranged from 0 to 300 minutes, with a median time of 10 minutes and a standard deviation of 46 minutes. Notably, no respondents reported treating drinking water. When households had stored water on premises at the time of our visit (n=98), the median water storage time was 6 hours.

Child Health
Overall, caregivers reported diarrhea in the last 7 days for over one-third of children (**Table S14**). Diarrheal prevalence peaked in the 3-5 and 6-11 month age groups. In our surveys we asked about diarrhea using two different definitions: 1) caregiver-defined diarrhea, 2) watery/soft stool and 3+ bowel movements in 24 hours. Diarrheal prevalence was similar between the two definitions. The overall prevalence of diarrhea in the last 7 days using each definition were 1) 36% and 2) 28%.

Food hygiene
For all 28 food samples collected, field staff recorded information on food storage and serving practices. Overall, 57% of food was cooled without a lid, and 64% was not reheated before serving. Food was frequently stored uncovered (46%) and on the ground (43%). Field staff observed the food preparation areas and found flies (54%), trash (43%), and animal feces (21%).

Discussion
We found a positive association between maternal prenatal antibiotic use and child infection. Prenatal antibiotic use can alter the maternal microbiome and, in turn, impact child microbiome and immune development after birth. Gut colonization begins in the womb, and disruptions of the maternal microbiome during pregnancy can alter the colonization period in the fetus.[2] Prior research has shown that mother's antibiotic use

before or during pregnancy is associated with infection-related hospitalizations in children.[3] Reducing environmental exposure to pathogens and subsequent morbidity during pregnancy could reduce unnecessary antibiotic use. Promoting antibiotic stewardship will also be vital in reducing unnecessary antibiotic use.[2]

Tables

Table S1: Primers and probes used in TaqMan Array Card

Type	Pathogen	Gene	Forward primer	Reverse primer	Probe	Source
Bacteria	Aeromonas	aha1	ACCGCTGCTCATTACTCTGATG	CCAACCCAGACGGGAAGAA	TGATGGTGAGCTGGTTG	6
	Campylobacter coli/jejuni	cadF	CTGCTAAACCATAGAAATAAAATT TCTCAC	CTTTGAAGGTAATTTAGATATGG ATAATCG	CATTTTGACGATTTTTGGCTTGA	7
	Campylobacter jejuni	hipO	CTTGCGGTCATGATGGACATAC	AGCACCACCCAAACCCTCTTCA	TGCTTGCTGCAAAGTATT	Levy
	C. coli	GlyA	AAACCAAAGCTTATCGTGTGC	AGTGCAGCAATGTGTGCAAT	TAAGCTCCAACTTCATCCG	Levy
	E. coli/ Shigella	ipaH	CCTTTTCCGCGTTCCTTGA	CGGAATCCGGAGGTATTGC	CGCCTTTCCGATACCGTCTCTGC A	7
	EAEC	aggR	GGAAGCAATACATATCTTAGAAA TGAACTC	TCGGACAACTGCAAGCATCTAC	TCCGTATATTATCATCAGGGCAT CCTTTAGGCGT	8
	EAEC	aaiC	ATTGTCCTCAGGCATTTCAC	ACGACACCCCTGATAAACAA	TAGTGCATACTCATCATTTAAG	7
	EAEC	aatA	CTGGCGAAAGACTGTATCAT	TTTTGCTTCATAAGCCGATAGA	TGGTTCTCATCTATTACAGACAG C	7
	Bacteroides fragilis	bft	GGGACAAGGATTCTACCAGCTTT ATA	ATTCGGCAATCTCATTCATCATT	CAATGGCGAATCCATCAG	9
	Clostridium difficile	tcdB	GGTATTACCTAATGCTCCAAATA G	TTTGTGCCATCATTTTCTAAGC	CCTGGTGTCCATCCTGTTTC	10
	EPEC	bfpA	TGGTGCTTGCGCTTGCT	CGTTGCGCTCATTACTTCTG	CAGTCTGCGTCTGATTCCAA	7
	EPEC	eae	CATTGATCAGGATTTTTCTGGTG ATA	CTCATGCGGAAATAGCCGTTA	ATACTGGCGAGACTATTTCAA	7
	EIEC	virF	TCTGAGGAGGAGGTTTCTATCGA TT	GAAACAGCTGATAAAAGGCAAGC T	CCGAAAGGCATCTCTTTT	Levy
	ETEC-LT	LT	TTCCCACCGGATCACCAA	CAACCTTGTGGTGCATGATGA	CTTGGAGAGAAGAACCCT	7
	ETEC-ST	estA	AAGCATGAATAGTAGCAATTACT GCT	TTAATAGCACCCGGTACAAGCA	AACAACACAATTCAC	11
	EHEC 0157H7	rfbE	TTTCACACTT ATTGGATGG TCTCAA	CGATGAGTTT ATCTGCAAG GTGAT	CTCTCTTTCCT CTGCGGTCCT	12
	Salmonella enterica	ttr	CTCACCAGGAGATTACAACATGG	AGCTCAGACCAAAAGTGACCATC	CACCGACGGCGAGACCGACTTT	13
	Salmonella typhi	tviB	TGTGGTAAAGGAACTCGGTAAA	GACTTCCGATACCGGGATAATG	TGGATGCCGAAGAGGTAAGACG AGA	Levy
	Salmonella typhi	STY0201	CGCGAAGTCAGAGTCGACATAG	AAGACCTCAACGCCGATCAC	CAGCCTGCTCCAGAACA	Levy
	Shiga-like toxin 1	stx1	CATCGCGAGTTGCCAGAAT	GCGTAATCCCACGGACTCTTC	CTGCCGGACACATAGAAGGAAA CTCATCA	14
	Shiga-like toxin 2	stx2	CCACATCGGTGTCTGTTATTAAC C	GGTCAAAACGCGCCTGATAG	TTGCTGTGGATATACGAGG	7
	Shigella flexneri 6	T3RE	CTTTCAACGCACGAATATCAAC	GAACCTGATCCAGACGGAGA	TTCTTCAGAACCGGGTTTTG	9

	Shigella flexneri 6	O-antigen	CTCCTATCCGTGATTATAGTGCA	GCACACACAACTCACTGTATTT	TCCTTCTCACGATTAAAATC	9
	Vibrio cholerae	toxR	GTTTGGCGAGAGCAAGGTTT	TCTCTTCTTCAACCGTTTCCA	CGCAGAGTCGAAATGGCTTGG	7
	E. coli	uidA	CGGAAGCAACGCGTAAACTC	TGAGCGTCGCAGAACATTACA	CGCGTCCGATCACCTGCGTC	15
	Helicobacter pylori	ureC	GACACCAGAAAAAGCGGCTA	AGCGCATGTCTTCGGTTAAA	TCACTAAAGCGTTTTCTACC	10
Viruses	Adenovirus	Hexon	GCCACGGTGGGGTTTCTAAACTT	GCCCCAGTGGTCTTACATGCACA TC	TGCACCAGACCCGGGCTCAG	7
	Adenovirus 40/41	Fiber	AACTTTCTCT CTTAATAGA CGCC	AGGGGGCTA GAAAACAAA A	CTGACACGGG CACTCT	12
	Astrovirus	Capsid	CAGTTGCTT GCTGCGTTC A	CTTGCTAGCC ATCACACTTC T	CACAGAAGA GCAACTCCAT CGC	12
	Enterovirus	5' UTR	CCCTGAATGCGGCTAATCC	GCGATTGTCACCATWAGCAG	CCGACTACTTTGGGWGTCCGT	Levy
	Norovirus GI	ORF1-ORF2	CGYTGGATG CGNTTYCAT GA	CTTAGACGCC ATCATCATTY AC	TGGACAGGAG ATCGC	12
	Norovirus GII	ORF1-ORF2	CARGARBCNATGTTYAGR TGGATGAG	TCGACGCCATCTTCATTCACA	TGGGAGGGCGATCGCAATCT	7
	Rotavirus A	NSP3	ACCATCTWCACRTRACCCTCTAT GAG	GGTCACATAACGCCCCTATAGC	AGTTAAAAGCTAACACTGTCAAA	7
Protozoa	Cryptosporidium spp.	18S	GGGTTGTATTTATTAGATAAAGA ACCA	AGGCCAATACCCTACCGTCT	TGACATATCATTCAAGTTTCTGAC	7
	C hominis	CH LIB 13	TCCTTGAAA TGAATATTTG TGACTCG	AAATGTGGT AGTTGCGGTT GAAA	CTTACTTCGTG GCGGCGT	12
	C parvum	CP LIB13	TCCTTGAAA TGAATATTTG TGACTCG	TTAATGTGGT AGTTGCGGTT GAAC	TATCTCTTCGT AGCGGCGTA	12
	Cyclospora cayetanesis	CC 18S	AAAAGCTCGTAGTTGGATTTCTG	AACACCAACGCACGCAGC	AAGGCCGGATGACCACGA	Levy
	Giardia duodenalis	18S	GACGGCTCAGGACAACGGTT	TTGCCAGCGGTGTCCG	CCCGCGGCGGTCCCTGCTAG	7
	E. histolytica	18S	ATTGTCGTGGCATCCTAACTCA	GCGGACGGCTCATTATAACA	TCATTGAATGAATTGGCCATTT	7
Helminths	Ascaris	ITS1	GTAATAGCAGTCGGCGGTTTCTT	GCCCAACATGCCACCTATTC	TTGGCGGACAATTGCATGCGAT	7
	Trichuris	18S	TTGAAACGACTTGCTCATCAACT T	CTGATTCTCCGTTAACCGTTGTC	CGATGGTACGCTACGTGCTTACC ATGG	7
	Necator americanus	ITS2	CTGTTTGTCGAACGGTACTTGC	ATAACAGCGTGCACATGTTGC	CTGTACTACGCATTGTATAC	13
	Ancyclostoma duodenale	ITS2	GAATGACAGCAAACTCGTTGTTG	ATACTAGCCACTGCCGAAACGT	ATCGTTTACCGACTTTAG	10
	Stongyloides stercoralis	Dispersed repetitive sequence	TCCAGAAAAGTCTTCACTCTCCA G	TGCGTTAGAATTTAGATATTATTG TTGCT	TCAGCTCCAGTTGAACAACAGCC TCCAA	10

Controls	16S	Total bacteria	TCCTACGGGAGGCAGCA	GGACTACCAGGGTATCTAATCCTG	CGTATTACCGCGGCTGCT	[7]
	CrAssphage	orf00024	CAGAAGTACAAACTCCTAAAAAACGTAGAG	GATGACCAATAAACAAGCCATTAGC	AATAACGATTTACGTGATGTAAC	[9]

Table S2: Standard curve equations and efficiencies

Pathogen	Target	Intercept	Estimate	R^2	Efficiency	LOQ (copies)	LOD (copies)
Aeromonas	aha1	36.39	-3.41	0.99	97	10	10
Campylobacter coli/jejuni	cadF	36.88	-3.53	0.99	92	10	10
Campylobacter jejuni	hipO	37.38	-3.51	0.99	93	10	10
C. coli	GlyA	36.64	-3.45	0.99	95	10	10
E. coli/ Shigella	ipaH	36.02	-3.43	0.99	96	10	10
EAEC	aggR	35.54	-3.35	0.96	99	10	10
EAEC	aaiC	35.36	-3.43	0.99	96	10	10
EAEC	aatA	34.94	-3.38	0.97	98	100	10
Bacteroides fragilis	bft	36.90	-3.49	0.99	93	10	10
Clostridium difficile	tcdB	35.51	-3.44	0.99	95	100	10
EPEC	bfpA	36.22	-3.42	1.00	96	10	10
EPEC	eae	36.58	-3.51	0.99	93	10	10
EIEC	virF	36.53	-3.57	0.99	90	10	10
ETEC-LT	LT	35.84	-3.51	1.00	93	10	10
ETEC-ST	estA	34.98	-3.35	0.99	99	100	10
EHEC 0157H7	rfbE	36.49	-3.65	1.00	88	10	10
Salmonella enterica	ttr	36.56	-3.51	0.99	93	10	10
Salmonella typhi	tviB	34.97	-3.43	1.00	96	100	10
Salmonella typhi	STY0201	37.78	-3.59	1.00	90	10	10
Shiga-like toxin 1	stx1	36.61	-3.46	0.99	95	10	10
Shiga-like toxin 2	stx2	36.11	-3.47	0.99	94	10	10
Shigella flexneri 6	T3RE	37.63	-3.69	0.98	87	10	10
Shigella flexneri 6	O-antigen	34.47	-3.33	0.92	100	10	10
Vibrio cholerae	toxR	36.16	-3.67	0.98	87	10	10
E. coli	uidA	37.11	-3.67	0.99	87	10	10

Helicobacter pylori	ureC	36.51	-3.44	1.00	95	100	10
Adenovirus	Hexon	37.62	-3.56	1.00	91	10	10
Adenovirus 40/41	Fiber	36.47	-3.64	0.99	88	10	10
Astrovirus	Capsid	37.44	-3.72	0.98	86	100	10
Enterovirus	5' UTR	35.97	-3.47	1.00	94	10	10
Norovirus GI	ORF1-ORF2	36.11	-3.60	0.99	90	10	10
Norovirus GII	ORF1-ORF2	36.84	-3.65	0.98	88	10	10
Rotavirus A	NSP3	36.53	-3.48	0.98	94	100	10
Cryptosporidium spp.	18S	36.98	-3.76	0.98	84	10	10
C hominis	CH LIB 13	37.22	-3.68	0.99	87	10	10
C parvum	CP LIB13	35.53	-3.50	0.99	93	100	10
Cyclospora cayetanesis	CC 18S	37.78	-3.72	0.98	86	10	10
Giardia duodenalis	18S	36.27	-3.59	0.99	90	10	10
E. histolytica	18S	36.77	-3.43	0.91	96	10	10
Ascaris	ITS1	36.82	-3.50	0.99	93	10	10
Trichuris	18S	37.48	-3.69	0.99	87	10	10
Necator americanus	ITS2	37.63	-3.46	1.00	94	10	10
Ancyclostoma duodenale	ITS2	37.41	-3.54	0.99	92	10	10
Stongyloides stercoralis	Dispersed repetitive sequence	36.60	-3.65	0.98	88	10	10

Table S3: Limit of detection by sample type

**Sample Type**	**LOD**
Feces (gene copies/g)	370
Soil (gene copies/g)	60
Drinking water (gene copies/100 mL)	110
Hand rinse (gene copies/100 mL)	110

**Table S4:** gBlock sequences
**Sequence 1:**<br>accgctgctcattactctgatgttgatggtgagctggttggttcttcccgtctgggttggctttgaaggtaatttagatatggataatcgttatgcaccagggattagacttggttatcattttgacgatttttggcttgatcaattagaatttgggttagagcattattctgatgttaaatatacaaatactaataaaactacagatattacaagaacttatttgagtgctattaaaggtattgatgtaggtgagaaattttatttctatggtttagcagcttgcggtcatgatggacatactacttctttattgcttgctgcaaagtatttagcaagtcagaattttaatggcactttaaatctttattttcaacctgctgaagagggtttgggtggtgctaaaccaaagcttatcgtgtgcggtgcgagtgcttatgctcgtattattgacttttcaaaatttagagagattgcggatgaagttggagcttatctttttgcagacattgcacacattgctgcactccttttccgcgttccttgaccgcctttccgataccgtctctgcacgcaatacctccggattccgttttggaagcaatacatatcttagaaatgaactcatatttcttgagagaggaataaatatatcagtaagattgcaaaagaagaaatcaacagtaaatccatttatcgcaatcagattaagcagcgatacattaagacgcctaaaggatgccctgatgataatatacggaatatcaaaagtagatgcttgcagttgtccgaattgtcctcaggcatttcacgctttttcaggaattgacggtactgtttttgatttaattaatttgaagcttagggttactaaacacatacaagaccttctggagaactttttaagagaggtgaaaaagaagttaaaatagaaattttacgtagggaatcaactaaatcaggtagtgcatactcatcatttaaggttgtttatcaggggtgtcgtctggcgaaagactgtatcattgataatttctttcagaaaagcatccagtttaattcttattctcttgatatcgaagagttagatattaataaacataacaatataaaaacgatgttaccagatataaatatagggttagggcagtatataaacaacaatcaatggttctcatctattacagacagccatttttatttatcattatcctataatcttctatcggcttatgaagcaaaagggacaaggattctaccagctttatactgggagatgagttcgcagtattacgtttttatcgcaatggcgaatccatcagctacatcgcatacaaggaagcgcaaatgatgaatgagattgccgaatggtattacctaatgctccaaatagagtatttgcttgggaaacaggatggacaccaggtttaagaagcttagaaaatgatggcacaaatggtgcttgcgcttgctgcaaccgttactgccggtgtgatgttttactaccagtctgcgtctgattccaataagtcacagaatgccatttcagaagtaatgagcgcaacgcattgatcaggatttttctggtgataatacccgtttaggtattggtggcgaatactggcgagactatttcaaaagtagcgttaacggctatttccgcatgagtccttgaaatgaatatttgtgactcgaaaaatttttagatttatttaatgtaactccagctgaattctttttcatgttctgcacaatgttcatttaactttttgtttgttttacgccgccacgaagtaagagataaattaataaattttcaaccgcaactaccacattt
**Sequence 2:**<br>ttcccaccggatcaccaagcttggagagaagaaccctggattcatcatgcaccacaaggttgttttaagcatgaatagtagcaattactgctgtgaattgtgttgtaatcctgcttgtaccgggtgctattaattttttcacacttattggatggtctcaattctaactaggaccgcagaggaaagagaggaattaaggaatcaccttgcagataaactcatcgttttctcaccaggagattacaacatggctaatttaacccgtcgtcagtggctaaaagtcggtctcgccgtcggtgggatggtcacttttggtctgagctttttgtggtaaaggaactcggtaaatatagttgtaaagtggatatttttgatccatgggtggatgccgaagaggtaagacgagagtatggcattatcccggtatcggaagtcttttcgcgaagtcagagtcgacataggcatagattttcaggccatacattaatttgccaaggttgctataaacatttgttctggagcaggctgacggaaattccgtgaactcgctggtgatcggcgttgaggtctttttcatcgcgagttgccagaatggcatctgatgagtttccttctatgtgtccggcagatggaagagtccgtgggattacgcttttccacatcggtgtctgttattaaccacaccccaccgggcagttattttgctgtggatatacgagggcttgatgtctatcaggcgcgttttgaccttttctttcaacgcacgaatatcaacgttggggttctggatcagcaccagcttcgccagctcatccagctcgttttgcgtgactggctcgccgttgcggattttcttcagaaccgggttttgctggaacagcggctccagcgccccctgaacctgctggcgatagagtttgaaatcaatcgagcggatattagttttgcgggtctccgtctggatcaggttcttttctcctatccgtgattatagtgcatatatatattatgaattgcagtcagtttatttttaaatcaacttggcgttattttatttactttgtttttattaattaatctccttctcacgattaaaatcataaaatacagtgagttgtgtgtgcttttgtttggcgagagcaaggttttgaagtcgatgattccagcttaacccaagccatttcgactctgcgcaaaatgctcaaagattcgacaaagtccccacaatacgtcaaaacggttccgaagcgtggttaccaattgatcgcccgagtggaaacggttgaagaagagattttcggaagcaacgcgtaaactcgacccgacgcgtccgatcacctgcgtcaatgtaatgttctgcgacgctcattttgacaccagaaaaagcggctatatggtagaaaacgctttagtgagcgctttaacttccataggctataatgtgattcaaatagggcctatgcccacccctgcgattgcgtttttaaccgaagacatgcgctttttgccacggtggggtttctaaacttgttccccaggctgaagtaggtatcggtggcacgggcgaactgcaccaggcccgggctcaggtactccgaggcgtcctgcccggagatgtgcatgtaagaccactggggcttttaactttctctcttaatagacgccccacttaatgctgacacgggcactcttcgccttcaaagtgctgcacctcttggactagtggacaaaacactaaaagttttgttttctagccccct
**Sequence 3:**<br>cagttgcttgctgcgttcatggcagaagatcatccttttaaagtgtatgtggaacactgcctgtcacggactgctaagcagcttcgtgattctggtctcccggccagactcacagaagagcaactccatcgcatttggaggggaggaccaaagaagtgtgatggctagcaagttttccctgaatgcggctaatcctaactgcggagcacatgccctcaacccagagggtagtgtgtcgtaacgggcaactctgcagcggaaccgactactttgggtgtccgtgtttccttttattcttacattggctgcttatggtgacaatcgcttttcgctggatgcgcttccatgatctgagcatgtggacaggggatcgcgatctcctgcccgattatgtaaatgatgatggcgtctaagttttaagagccaatgttca

```
gatggatgaggtgggagggcgatcgcaatcttgtgaatgaagatggcgtcgattttaccatcttcacgtaaccctctatgagcacaatagttaaaagctaacactgt
caaaaacctaaatggctataggggcgttatgtgaccttttgggttgtatttattagataaagaaccaatataattggtgactcataataactttacggatcactttaaatg
tgacatatcattcaagtttctgacctatcagctttagacggtagggtattggcctttttctgaggaggaggtttctatcgatttgttcaaatctataaaagagatgcctttcg
gcaaaagaaagatctatagtttagcttgccttttatcagctgtttctttttccttgaaatgaatatttgtgactcgaaaaatttttagatttatttaatgtaactccagctgaatt
ctttttcatgttctgcacaatgttcatttaactttttgtttgttttacgccgctacgaagagataaattaataaatgttcaaccgcaactaccacattaattttaaaagctcgta
gttggatttctgtcgtggtcatccggccttgcccgtagggtgtgcgcctgggttgcccgcggctttttccggtagccttccgcgcttcgctgcgtgcgttggtgtttttgac
ggctcaggacaacggttgcaccccccgcggcggtccctgctagccggacaccgctggcaattttattgtcgtggcatcctaactcacttagaatgtcatttctcaatt
cattgaatgaattggccattttgtactaatacaaactggatcgtctcaagtattatctttatcattcacaaagctatccttgtattttactaaccaaagaaactattactgtta
taatgagccgtccgcttttgtaatagcagtcggcggtttctttttttttggcggacaattgcatgcgatttgctatgtgttgagggagaataggtggcatgttgggctttttg
aaacgacttgctcatcaactttcgatggtacgctacgtgcttaccatggtgacaacggttaacggagaatcagttttctgtttgtcgaacggtacttgctctgtactacg
cattgtatacgtgttcagcaattcccgtttaagtgaagaacacacgtgcaacatgtgcacgctgttatttttgaatgacagcaaactcgttgttgctgctgaatcgttcac
cgactttagaacgtttcggcagtggctagtatttttccagaaaagtcttcactctccagattcagctccagttgaacaacagcctccaaaacagcaacaataatatct
aaattctaacgca
```

Table S5. Household enrollment by age group and subcounty, including number of households enrolled in longitudinal data collection.

	Overall		Turkana South		Samburu North	
**Age Group**	**Total**	**Longitudinal**	**Total**	**Longitudinal**	**Total**	**Longitudinal**
All	*100*	*61*	*50*	*30*	*50*	*31*
0-2 months	24	24	12	12	12	12
3-5 months	22	21	11	10	11	11
6-11 months	30	16	15	8	15	8
12-23 months	24	0	12	0	12	0

Table S6. Samples collected by type.

**Sample Type**	**Total**	***E. coli* measured by culture**
Child Stool	162	0
Mother/caregiver stool	60	0
Animal feces	238	0
Soil	163	73
Food	40	10
Hand rinse	92	49
Drinking water (stored)	116	61

Table S7. Detection of pathogens by sample type

		Camel Feces	Cattle Feces	Poultry Feces	Food	Child Hands	Child Stool	Dog Feces	Drinking Water	Goat Feces	Soil	Mother Stool	Sheep Feces
**Bacteria**	Aeromonas	0 / 3	1 / 45	0 / 55	0 / 40	6 / 92	0 / 162	0 / 13	5 / 116	0 / 57	1 / 163	0 / 60	0 / 65
	Bacteroides fragilis	1 / 3	2 / 45	7 / 55	0 / 40	10 / 92	19 / 162	6 / 13	2 / 116	4 / 57	18 / 163	6 / 60	9 / 65
	Campylobacter coli/jejuni	0 / 3	1 / 45	14 / 55	0 / 40	1 / 92	16 / 162	0 / 13	0 / 116	4 / 57	17 / 163	3 / 60	3 / 65
	Clostridium difficile	0 / 3	0 / 45	0 / 55	0 / 40	0 / 92	2 / 162	1 / 13	0 / 116	0 / 57	0 / 163	0 / 60	0 / 65
	E. coli/ Shigella	1 / 3	0 / 45	3 / 55	1 / 40	11 / 92	3 / 162	3 / 13	1 / 116	0 / 57	11 / 163	13 / 60	0 / 65
	EAEC	0 / 3	0 / 45	26 / 55	0 / 40	13 / 92	91 / 162	7 / 13	0 / 116	8 / 57	68 / 163	37 / 60	10 / 65
	EHEC 0157H7	0 / 3	0 / 45	3 / 55	0 / 40	2 / 92	3 / 162	0 / 13	0 / 116	0 / 57	4 / 163	2 / 60	1 / 65
	EIEC	1 / 3	0 / 45	3 / 55	0 / 40	1 / 92	0 / 162	2 / 13	0 / 116	0 / 57	3 / 163	11 / 60	0 / 65
	EPEC	0 / 3	2 / 45	32 / 55	2 / 40	22 / 92	59 / 162	9 / 13	8 / 116	13 / 57	61 / 163	30 / 60	15 / 65
	ETEC-LT	0 / 3	1 / 45	18 / 55	2 / 40	42 / 92	24 / 162	4 / 13	14 / 116	6 / 57	59 / 163	17 / 60	6 / 65
	ETEC-ST	0 / 3	0 / 45	7 / 55	0 / 40	25 / 92	3 / 162	2 / 13	4 / 116	2 / 57	25 / 163	3 / 60	1 / 65
	Helicobacter pylori	0 / 3	0 / 45	0 / 55	0 / 40	2 / 92	10 / 162	0 / 13	0 / 116	0 / 57	0 / 163	6 / 60	0 / 65
	STEC	1 / 3	10 / 45	19 / 55	1 / 40	17 / 92	12 / 162	6 / 13	7 / 116	29 / 57	19 / 163	8 / 60	34 / 65
	Salmonella enterica	0 / 3	0 / 45	2 / 55	0 / 40	2 / 92	1 / 162	0 / 13	2 / 116	0 / 57	7 / 163	0 / 60	0 / 65
	Salmonella typhi	0 / 3	0 / 45	0 / 55	0 / 40	0 / 92	0 / 162	0 / 13	0 / 116	0 / 57	3 / 163	0 / 60	0 / 65
	Shigella flexneri 6	0 / 3	0 / 45	0 / 55	0 / 40	0 / 92	1 / 162	0 / 13	0 / 116	0 / 57	0 / 163	2 / 60	0 / 65
	Vibrio cholerae	0 / 3	0 / 45	0 / 55	1 / 40	5 / 92	0 / 162	0 / 13	1 / 116	0 / 57	27 / 163	1 / 60	0 / 65
**Helminths**	Ancyclostoma duodenale	0 / 3	0 / 45	0 / 55	0 / 40	0 / 92	0 / 162	0 / 13	0 / 116	0 / 57	0 / 163	0 / 60	0 / 65
	Ascaris	0 / 3	0 / 45	0 / 55	0 / 40	0 / 92	0 / 162	0 / 13	0 / 116	0 / 57	0 / 163	0 / 60	0 / 65
	Necator americanus	0 / 3	0 / 45	0 / 55	0 / 40	0 / 92	0 / 162	0 / 13	0 / 116	0 / 57	0 / 163	0 / 60	0 / 65
	Stongyloides stercoralis	0 / 3	0 / 45	0 / 55	0 / 40	0 / 92	0 / 162	0 / 13	0 / 116	0 / 57	0 / 163	0 / 60	0 / 65
	Trichuris	1 / 3	1 / 45	0 / 55	0 / 40	0 / 92	0 / 162	0 / 13	0 / 116	7 / 57	5 / 163	0 / 60	7 / 65
**Protozoa**	C hominis	0 / 3	0 / 45	0 / 55	0 / 40	0 / 92	3 / 162	0 / 13	0 / 116	0 / 57	0 / 163	0 / 60	0 / 65
	C parvum	0 / 3	0 / 45	0 / 55	0 / 40	0 / 92	0 / 162	0 / 13	0 / 116	0 / 57	0 / 163	0 / 60	0 / 65
	Cryptosporidium spp.	0 / 3	17 / 45	13 / 55	0 / 40	8 / 92	6 / 162	5 / 13	14 / 116	10 / 57	57 / 163	1 / 60	10 / 65
	Cyclospora cayetanesis	0 / 3	0 / 45	0 / 55	0 / 40	2 / 92	0 / 162	1 / 13	0 / 116	2 / 57	5 / 163	0 / 60	3 / 65
	E. histolytica	0 / 3	0 / 45	0 / 55	0 / 40	0 / 92	0 / 162	0 / 13	0 / 116	0 / 57	0 / 163	0 / 60	0 / 65
	Giardia duodenalis	0 / 3	5 / 45	15 / 55	18 / 40	68 / 92	32 / 162	9 / 13	53 / 116	11 / 57	69 / 163	16 / 60	12 / 65
**Viruses**	Adenovirus 40/41	0 / 3	0 / 45	3 / 55	0 / 40	14 / 92	24 / 162	4 / 13	6 / 116	0 / 57	8 / 163	5 / 60	2 / 65
	Astrovirus	0 / 3	0 / 45	2 / 55	0 / 40	3 / 92	15 / 162	0 / 13	0 / 116	0 / 57	4 / 163	0 / 60	0 / 65
	Enterovirus	0 / 3	0 / 45	15 / 55	0 / 40	11 / 92	76 / 162	3 / 13	0 / 116	2 / 57	25 / 163	14 / 60	3 / 65
	Norovirus GI	0 / 3	0 / 45	0 / 55	0 / 40	0 / 92	8 / 162	0 / 13	0 / 116	0 / 57	1 / 163	1 / 60	0 / 65
	Norovirus GII	0 / 3	0 / 45	4 / 55	0 / 40	3 / 92	20 / 162	5 / 13	0 / 116	0 / 57	3 / 163	4 / 60	0 / 65
	Rotavirus A	0 / 3	0 / 45	1 / 55	0 / 40	5 / 92	4 / 162	0 / 13	1 / 116	0 / 57	3 / 163	0 / 60	0 / 65
**E. coli**	E. coli by culture				14 / 33	50 / 107			84 / 111		66 / 111		

Table S8. Odds ratios of child diarrhea given pathogen detection in child stool. Bold indicates statistical significance ($p < 0.05$).

**Pathogen**	**Odds Ratio (95% CI)**	**p**
Adenovirus 40/41	0.71 (0.29, 2.32)	0.67
Astrovirus	2.05 (0.79, 7.36)	0.15
C hominis	0 (0.02, 7.01)	0.37
Campylobacter coli/jejuni	1.06 (0.42, 3.68)	0.75
Clostridium difficile	0 (0.02, 10.66)	0.52
Cryptosporidium spp.	1.28 (0.35, 9.81)	0.57
E. coli/ Shigella	0.84 (0.2, 12.03)	0.82
EAEC	0.56 (0.29, 1.21)	0.16
EHEC 0157H7	0 (0.02, 7.01)	0.37
EPEC	0.62 (0.31, 1.4)	0.28
ETEC-LT	0.6 (0.23, 2.15)	0.50
ETEC-ST	2.61 (0.57, 34.58)	0.21
Enterovirus	0.85 (0.45, 1.85)	0.79
Giardia duodenalis	0.85 (0.39, 2.29)	0.87
Helicobacter pylori	1.29 (0.42, 6.69)	0.55
Norovirus GI	0.41 (0.11, 4.29)	0.60
Norovirus GII	0.41 (0.15, 1.67)	0.21
**Rotavirus A**	**11.08 (1.33, 482.08)**	**0.01**
STEC	1.29 (0.46, 5.39)	0.53
Salmonella enterica	2.57 (0.31, 196.41)	0.28
Shigella flexneri 6	0.0 (0.03, 21.11)	0.72

Table S9. Prevalence of child diarrhea in the last 7 days.

Age Group	N	Diarrhea last 7 days: Caregiver definition	Proportion with diarrhea: Caregiver definition	Proportion with diarrhea: WHO definition
All	100	36	0.36	0.28
0-2 months	24	7	0.29	0.17
3-5 months	22	10	0.45	0.41
6-11 months	30	11	0.37	0.27
12-23 months	24	8	0.33	0.33

Table S10. Correlations between pathogens and total E. coli measured by TAC (*uidA* gene): *r* correlation, p value.

Pathogen	Soil	Food	Hands	Drinking water	Chicken feces	Goat feces	Sheep feces	Child stool
Adenovirus 40/41	0.05, 0.94					0.23, 0.94	-0.16, 0.94	
Astrovirus	0.39, 0.66				-0.1, 0.94			-0.18, 0.94
Clostridium difficile								-0.09, 0.94
Cryptosporidium spp.	-0.08, 0.94		0.07, 0.94		-0.08, 0.94			-0.01, 0.94
Cyclospora cayetanesis	-0.07, 0.94				**0.78, 0.00**			0.26, 0.37
E. coli/ Shigella	0.06, 0.94		0.45, 0.21					
E. histolytica	-0.17, 0.94							0.49, 0.21
Enterovirus	0.36, 0.94							
Giardia duodenalis	0.08, 0.94		**0.68, 0.00**	**1, 0.00**	0.22, 0.94			-0.1, 0.94
Rotavirus A	-0.07, 0.94		-0.18, 0.94		-0.1, 0.94	**0.71, 0.00**	-0.05, 0.94	0.45, 0.21
Salmonella typhi	0.01, 0.94	-0.18, 0.94	0.01, 0.94	0.1, 0.94	0.02, 0.94	0.05, 0.94	-0.21, 0.94	-0.08, 0.94
Stongyloides stercoralis	0.07, 0.94							

Table S11. Correlations between pathogens measured by TAC and total *E. coli* measured in culture; correlations between detection (right) and quantities (left). Displayed is the Pearon's r, p value adjusted for multiple comparisons using the Bonferroni adjustment. Bold indicates statistical significant ($p < 0.05$)

	Positive/negative				Quantities			
**Pathogen**	**Soil**	**Food**	**Hands**	**Drinking water**	**Soil**	**Food**	**Hands**	**Drinking water**
Adenovirus 40/41	0.13, 0.72		-0.04, 0.93	0.18, 0.72	0.17, 0.91			
Aeromonas	0.09, 0.72		0.1, 0.80	-0.04, 0.90	-0.08, 0.91		0.25, 0.91	-0.03, 0.92
Ancyclostoma duode le					-0.05, 0.91			
Ascaris								
Astrovirus			-0.13, 0.72				-0.04, 0.91	
C hominis								
C parvum								
Campylobacter coli/jejuni	-0.02, 0.94		-0.13, 0.72		-0.04, 0.91		0.18, 0.91	-0.06, 0.91
Clostridium difficile								
Cryptosporidium spp.	0.18, 0.72		0.19, 0.72	-0.05, 0.90				
Cyclospora cayetanesis	-0.07, 0.80		0.32, 0.72		-0.08, 0.91		0.34, 0.32	
E. coli/ Shigella	0.13, 0.72		0.28, 0.72		0.10, 0.91		0.41, 0.13	
E. histolytica								
EAEC	0.33, 0.34		-0.09, 0.80		0.15, 0.91		0.21, 0.91	
EHEC 0157H7	0.06, 0.81							
EIEC	0.16, 0.72		0.22, 0.72		-0.08, 0.91		**0.47, 0.05**	
EPEC	0.13, 0.72	-0.35, 0.72	-0.09, 0.80	0.09, 0.80	-0.04, 0.91		0.11, 0.91	
ETEC-LT	0.18, 0.72	0.29, 0.72	-0.04, 0.93	0.08, 0.80	0.01, 0.96	-0.15, 0.91	0.09, 0.91	**0.56, 0.00**
ETEC-ST	0.08, 0.78		-0.14, 0.72	-0.25, 0.72	-0.06, 0.91		-0.07, 0.91	-0.06, 0.91
Enterovirus	0.12, 0.72		-0.14, 0.72		0.03, 0.91		-0.07, 0.91	
Giardia duodenalis	0.13, 0.72	-0.35, 0.72	-0.17, 0.72	-0.15, 0.72	-0.04, 0.91	-0.24, 0.91	-0.08, 0.91	-0.02, 0.92
Helicobacter pylori			-0.13, 0.72				-0.04, 0.91	
Necator americanus								
Norovirus GI								
Norovirus GII	0.09, 0.72		-0.02, 0.95		**0.42, 0.01**		**0.47, 0.05**	
Rotavirus A	0.01, 0.95		0.32, 0.72		0.03, 0.91		0.02, 0.94	
STEC	-0.01, 0.96		0.00, 0.98	0.05, 0.90	0.05, 0.91			
Salmonella enterica			-0.13, 0.72				-0.04, 0.91	
Salmonella typhi	-0.05, 0.86				-0.05, 0.91			
Shigella flexneri 6								
Stongyloides stercoralis								
Trichuris	-0.16, 0.72				-0.05, 0.91			
Vibrio cholerae	0.23, 0.72		-0.13, 0.72		-0.07, 0.91		-0.04, 0.91	

Table S12. Results (Cq values) of inhibition testing on animal feces, human stool, and soil samples. Only targets detected within samples are displayed.

**Sample**	**Goat feces**		**Human stool**		**Soil 1**		**Soil 2**				**Soil 3**			
**Dilution**	**1**	**1:10**	**1**	**1:10**	**1**	**1:10**	**1**	**1:10**	**1:20**	**1:50**	**1**	**1:10**	**1:20**	**1:50**
CADF							32	36			46			
HIPO							32	34						
GLYA											35			
IPAH							36				39			
AGGR					38		34				34			
AAIC			32		33									
AATA					40	37	36				37			
BFT							34	48						
18s	17	19	16	18	12	12	14	17	18	20	13	16	18	19
BFPA			30	34										
EAE			32	35	33	35	29	32	33	35	36			
LT					34		33	36			35			
ESTA							34				30	35	32	
RFBE							29	33	33	36				
STX1	33	36												
STX2	31	33	35				31	33	34	36				
T3RE					40									
TOXR							29	31	33					
UIDA	26	30	26	29	27	30	25	28	29	31	30	33	35	
UREC			39											
HEXON							35							
5_UTR							32	38	44					
NGI_ORF1-ORF2							35		36					
NSP3							33	36	34					
CP_18S	35	40			22	21	26	28	29	32	28	31	32	34
CC_18S		38			36	33	34	35	36	40	41	40		
G_18S					29	29	30	35	32		30	33		
synthetic construct	33		33	36	34		32		36		33	35	36	
ORF00024	8	11	5	7	5	5	5	8	9	12	5	10	12	14

Figures

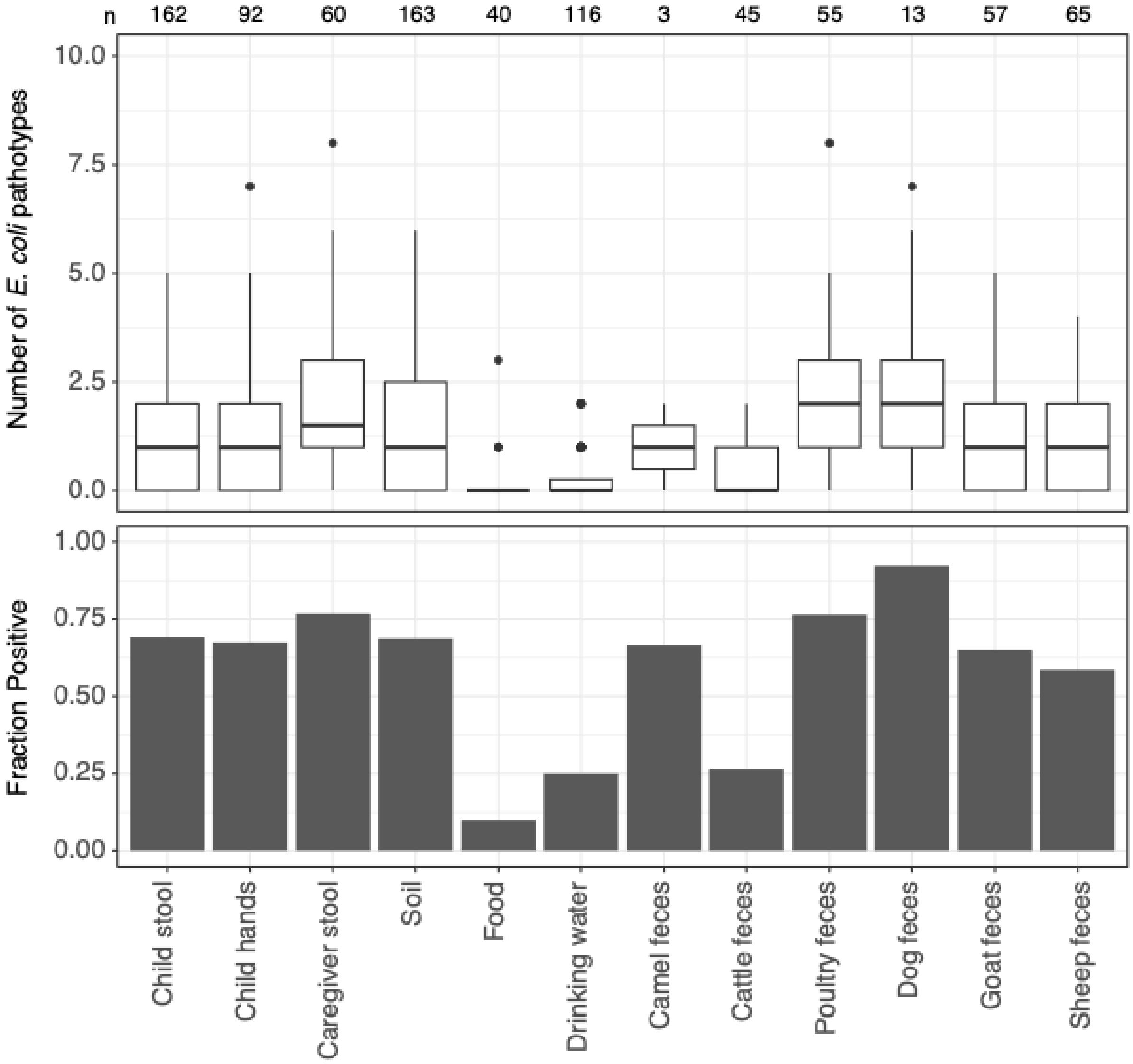

Figure S1. *E. coli* TAC detections by pathotype and sample. Top shows a boxplot of the number of pathotypes present in each sample. Bottom displays the fraction of samples positive for any *E. coli* pathotype by sample type.

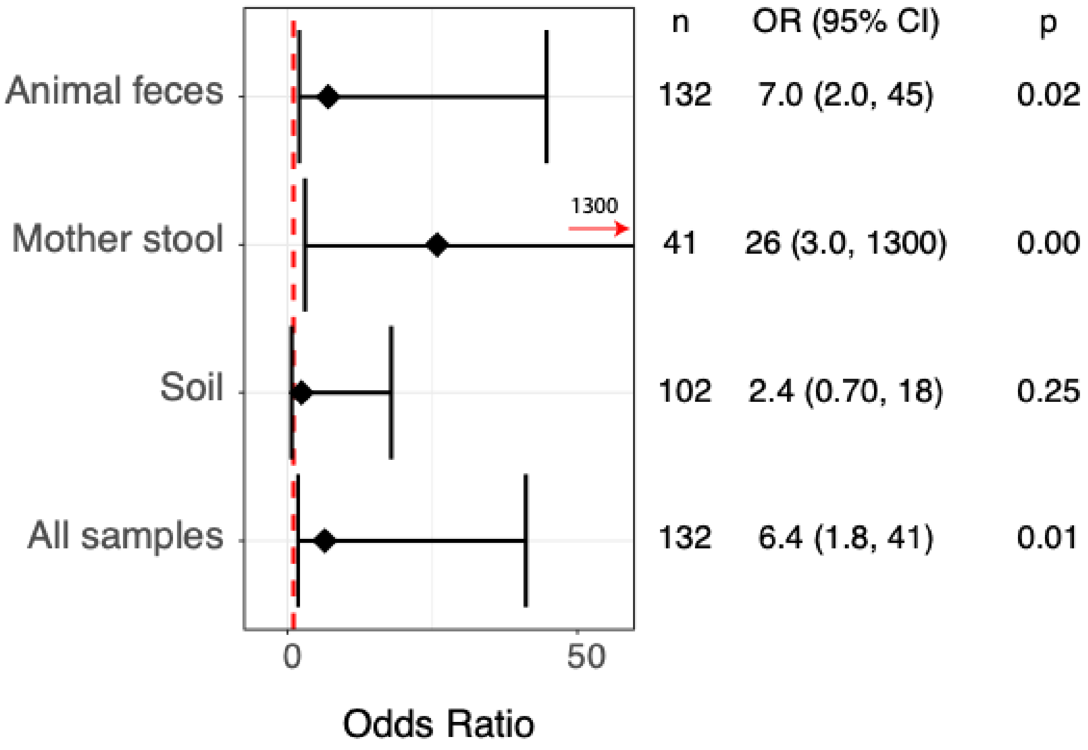

Figure S2: Odds ratios of pathogen detection in animal feces or environmental samples and subsequent or cooccurring new infection (both are included) with the same pathogen in children. Bars represent 95% confidence intervals. The dotted red line indicates an odds ratio of 1. The environmental samples category includes hands, soil, water, and food. Food alone is not displayed because of uncertainty around the point estimate.

Figure S3. Pathogen Transmission Graphs

Graphs of pathogen transmission for pathogen detection lagged one visit in blue, and co-detection in the same sampling visit to a household in red; the arrow direction indicates the sample type associated with detection in the other sample type on the prior visit. Circles are sample types; arrows are the odds ratios of pathogen co-occurrence between pairs. Only lines for odds ratios with a p-value < 0.05 are presented. Arrow weights correspond to the value of the odds ratio; a thicker edge indicates greater odds of co-occurrence.

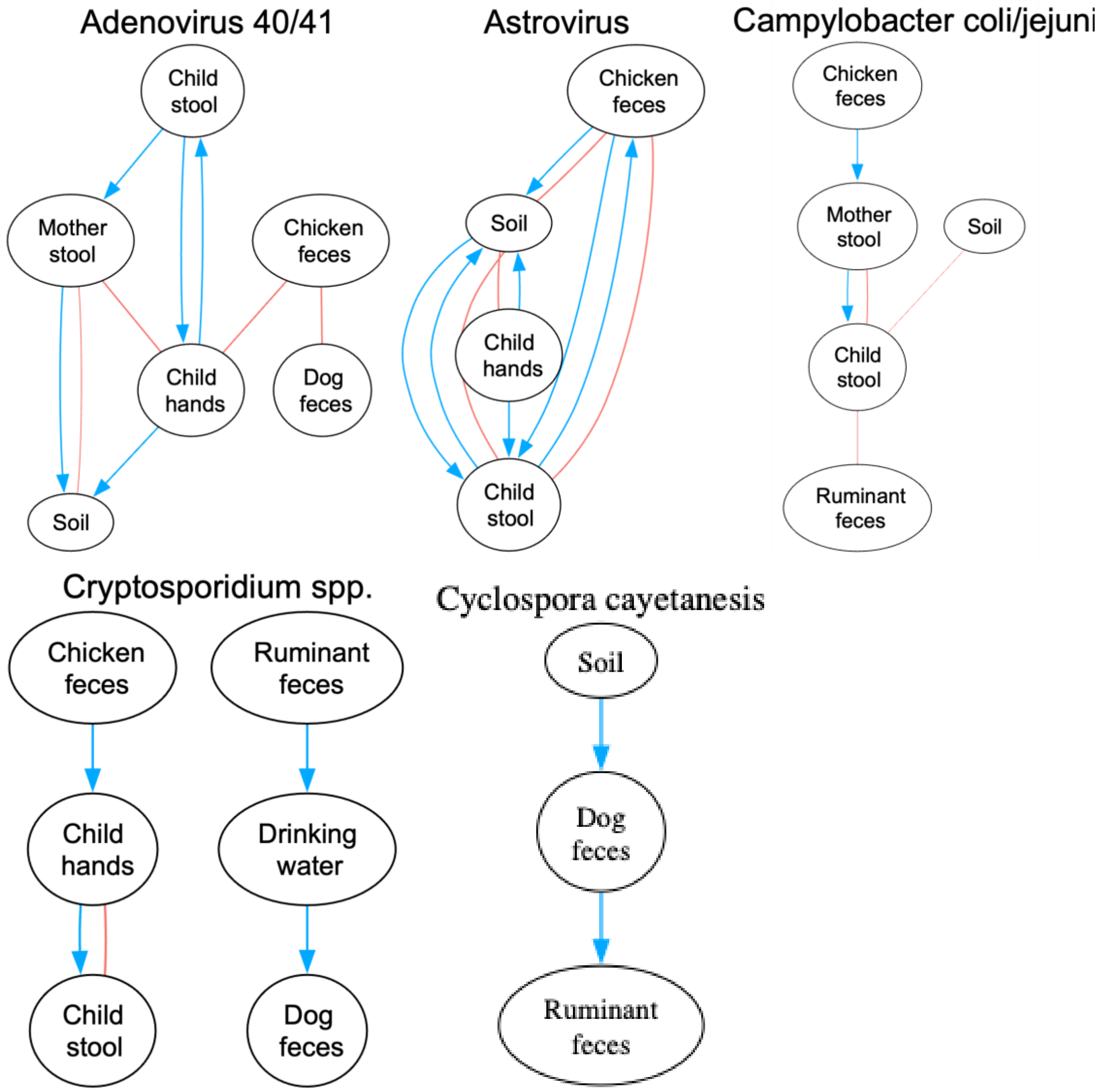

Enteroinvasive E. coli/Shigella

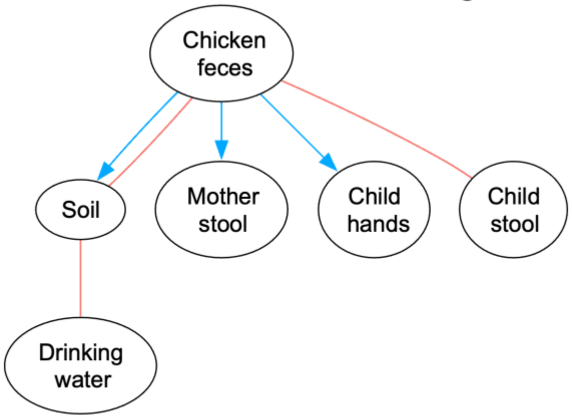

Enterohemorrhagic E. coli O157:H7

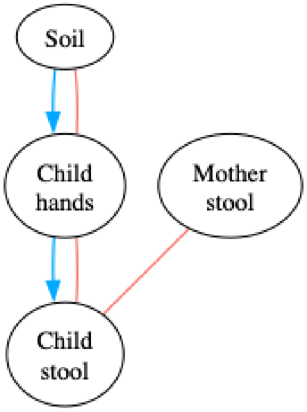

Enteroinvasive E. coli

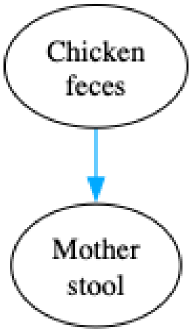

Enteroaggregative E. coli

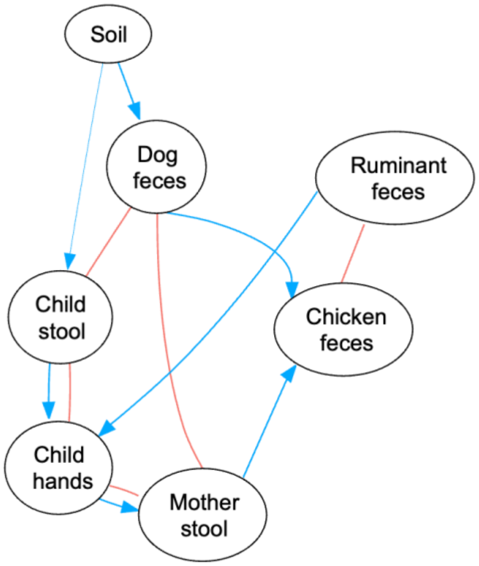

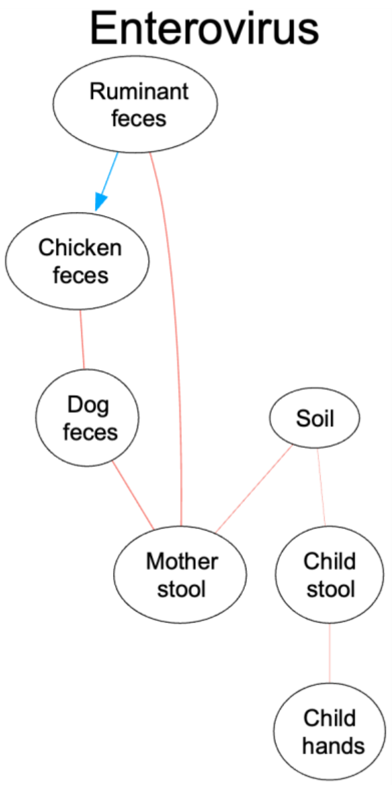
Enterovirus
Ruminant feces
Chicken feces
Dog feces
Soil
Mother stool
Child stool
Child hands

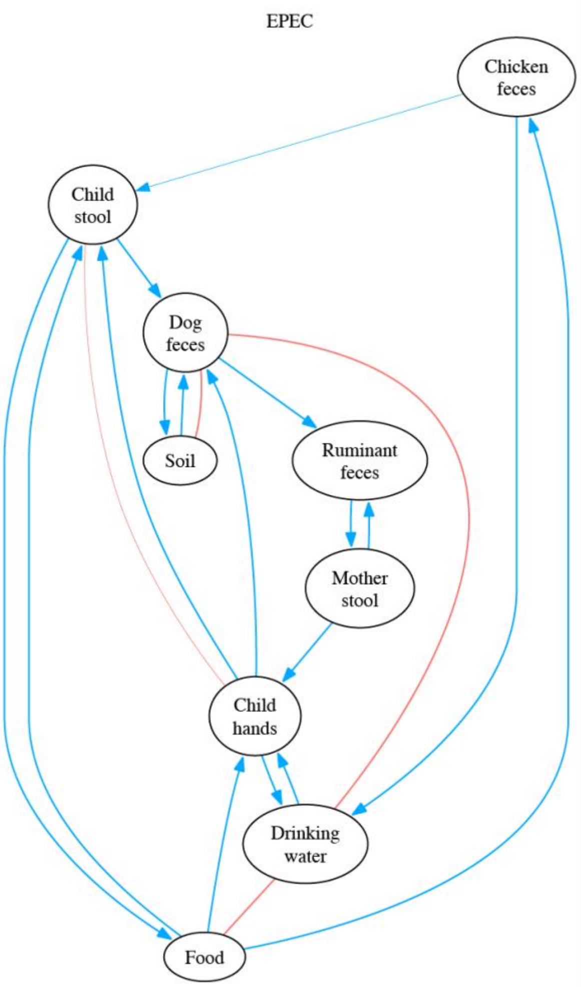
EPEC
Chicken feces
Child stool
Dog feces
Soil
Ruminant feces
Mother stool
Child hands
Drinking water
Food

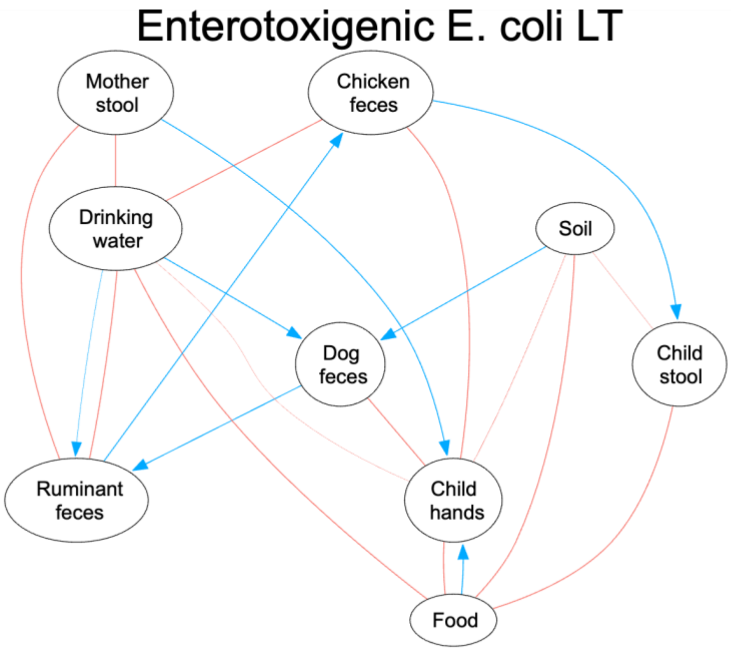
Enterotoxigenic E. coli LT
Mother stool
Chicken feces
Drinking water
Soil
Dog feces
Child stool
Ruminant feces
Child hands
Food

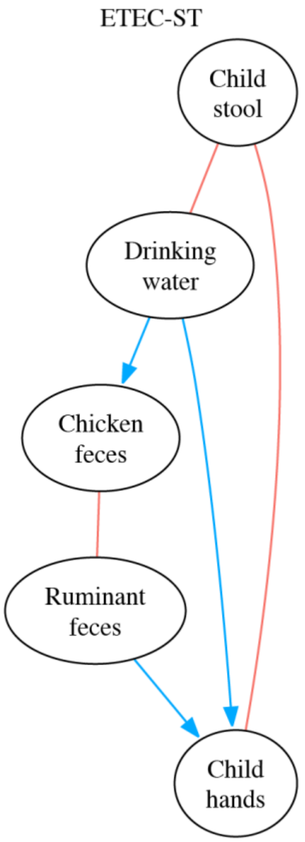
ETEC-ST
Child stool
Drinking water
Chicken feces
Ruminant feces
Child hands

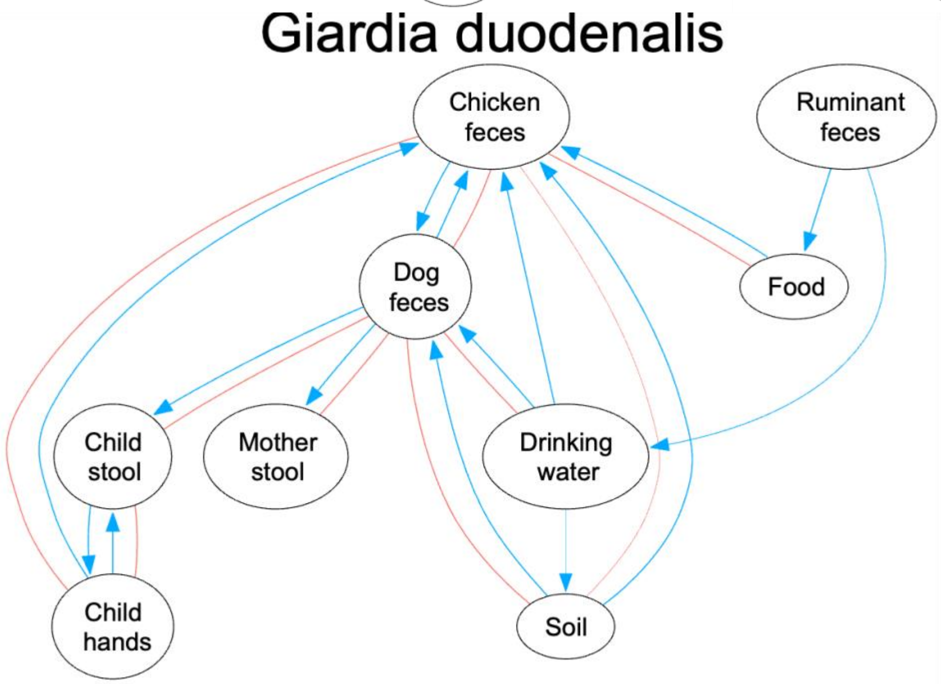
Giardia duodenalis
Chicken feces
Ruminant feces
Dog feces
Food
Child stool
Mother stool
Drinking water
Child hands
Soil

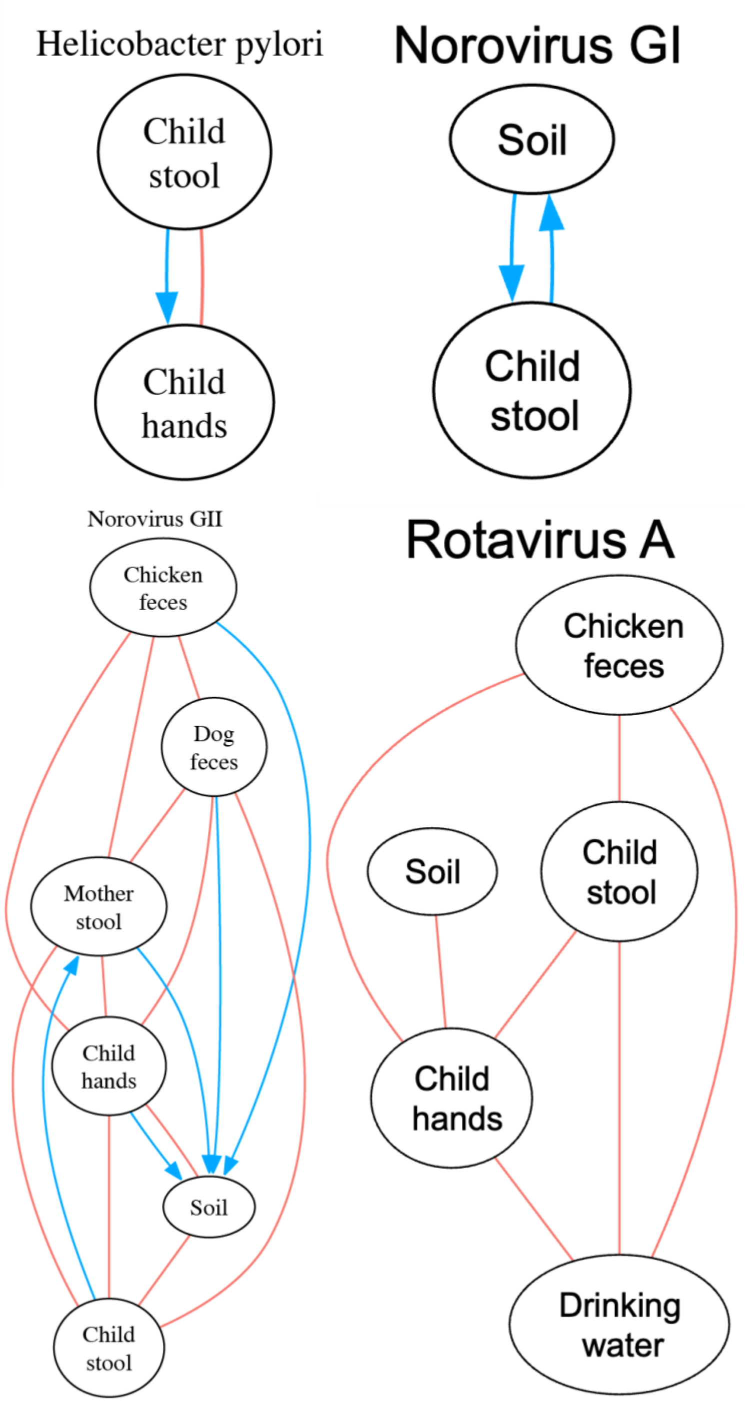
Helicobacter pylori
Child stool
Child hands
Norovirus GI
Soil
Child stool
Norovirus GII
Chicken feces
Dog feces
Mother stool
Child hands
Soil
Child stool
Rotavirus A
Chicken feces
Soil
Child stool
Child hands
Drinking water

STEC

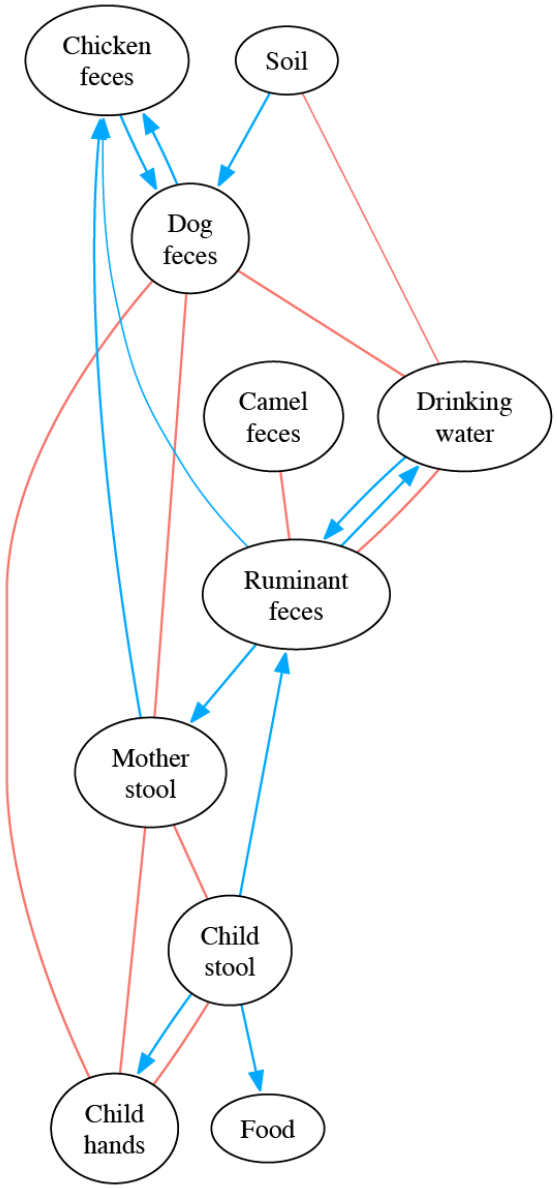
Chicken feces
Soil
Dog feces
Camel feces
Drinking water
Ruminant feces
Mother stool
Child stool
Child hands
Food

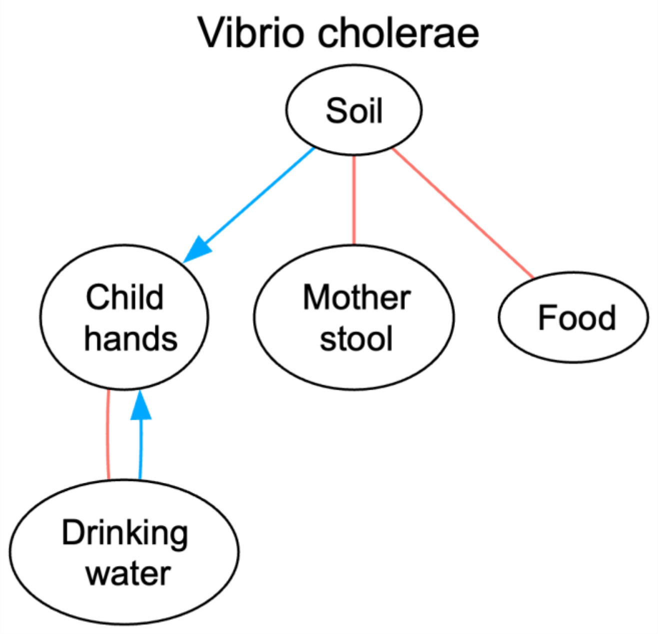
Vibrio cholerae
Soil
Child hands
Mother stool
Food
Drinking water

References

(1) IPA; Poverty Probability Index. *Kenya 2015 PPI*; 2018.

(2) Sulis, G.; Adam, P.; Nafade, V.; Gore, G.; Daniels, B.; Daftary, A.; Das, J.; Gandra, S.; Pai, M. Antibiotic Prescription Practices in Primary Care in Low- and Middle-Income Countries: A Systematic Review and Meta-Analysis. *PLOS Med.* **2020**, *17* (6), e1003139. https://doi.org/10.1371/journal.pmed.1003139.

(3) Miller, J. E.; Wu, C.; Pedersen, L. H.; de Klerk, N.; Olsen, J.; Burgner, D. P. Maternal Antibiotic Exposure during Pregnancy and Hospitalization with Infection in Offspring: A Population-Based Cohort Study. *Int. J. Epidemiol.* **2018**, *47* (2), 561–571. https://doi.org/10.1093/ije/dyx272.
